# The effect of a temperature‐sensitive prophage on the evolution of virulence in an opportunistic bacterial pathogen

**DOI:** 10.1111/mec.16638

**Published:** 2022-09-17

**Authors:** Matthieu Bruneaux, Roghaieh Ashrafi, Ilkka Kronholm, Elina Laanto, Anni‐Maria Örmälä‐Tiznado, Juan A. Galarza, Chen Zihan, Mruthyunjay Kubendran Sumathi, Tarmo Ketola

**Affiliations:** ^1^ Department of Biological and Environmental Science University of Jyväskylä Jyväskylä Finland; ^2^ Molecular and Integrative Biosciences Research Programme, Faculty of Biological and Environmental Sciences University of Helsinki Helsinki Finland; ^3^ Department of Laboratory Medicine Karolinska Institute Stockholm Sweden; ^4^ Shenzhen Research Institute The Chinese University of Hong Kong Shenzhen China; ^5^ Department of Ecology and Evolutionary Biology University of Arizona Tucson Arizona USA

**Keywords:** epigenetics, experimental evolution, opportunistic pathogen, prophage induction

## Abstract

Viruses are key actors of ecosystems and have major impacts on global biogeochemical cycles. Prophages deserve particular attention as they are ubiquitous in bacterial genomes and can enter a lytic cycle when triggered by environmental conditions. We explored how temperature affects the interactions between prophages and other biological levels using an opportunistic pathogen, the bacterium *Serratia marcescens*, which harbours several prophages and that had undergone an evolution experiment under several temperature regimes. We found that the release of one of the prophages was temperature‐sensitive and malleable to evolutionary changes. We further discovered that the virulence of the bacterium in an insect model also evolved and was positively correlated with phage release rates. We determined through analysis of genetic and epigenetic data that changes in the bacterial outer cell wall structure possibly explain this phenomenon. We hypothezise that the temperature‐dependent phage release rate acted as a selection pressure on *S. marcescens* and that it resulted in modified bacterial virulence in the insect host. Our study system illustrates how viruses can mediate the influence of abiotic environmental changes to other biological levels and thus be involved in ecosystem feedback loops.

## 1 INTRODUCTION

Viruses in general, and bacteriophages in particular, are increasingly being recognized as key actors of global biogeochemical cycles (Shelford & Suttle, 2018; Suttle, 2007; Weitz et al., 2015). They interact with global climate change via multiple feedback loops (Danovaro et al., 2011). Bacteriophages are viruses that can infect prokaryotes and are divided into two main categories based on their life cycle (Clokie et al., 2011). Lytic phages infect a host, reproduce within it and kill it upon release of new phage particles by cell lysis. Temperate phages, on the other hand, can switch between a lytic cycle and a facultative lysogenic cycle where they integrate into their host's genome as prophages and are carried over to the next generations, until a potential switch to a lytic life cycle triggered by the host physiological status, environmental conditions or stochastic effects (Howard‐Varona et al., 2017). The existence of those two life strategies (lytic and temperate) allows for a great variety of evolutionary interactions between phages and their hosts, encompassing both the usual richness of predator–prey (or parasite–host) eco‐evolutionary dynamics (Bohannan & Lenski, 2000) and the unique consequences arising from the existence of an integrated prophage in the lysogenic phase (e.g. Feiner et al., 2015). Prophages are ubiquitous in bacteria (Touchon et al., 2016; Tuttle & Buchan, 2020) and affect the evolution of bacterial genomes, ranging from mutualistic parasites to domesticated phages (Bobay et al., 2014; Breitbart et al., 2018). Because the relations between prophages and their microbial hosts constitute a major evolutionary force, it is crucial to understand how changes in their interactions are transmitted and amplified in higher‐level biological processes (Buck & Ripple, 2017; Zarnetske et al., 2012).

Prophages can affect their hosts in very direct ways (Argov et al., 2017). For example, the integration of a prophage into a host genome can alter bacterial gene regulation (Laumay et al., 2019; Schroven et al., 2021) or provide new virulence factors to the bacterium (Fortier & Sekulovic, 2013). Environmental conditions affect the relationship between prophages and bacteria in complex ways: temperature, nutrient abundance, the presence of abiotic stressors or even population densities can induce changes in bacterial physiological state that influence the balance between lytic and lysogenic cycles (Maurice et al., 2010, 2013). Conversely, integrated prophages switching from lysogenic to lytic cycle can have an immediate impact on bacterial virulence (Plunkett et al., 1999) and on bacterial communities, resulting in rapid and dramatic effects on ecosystems (Brum et al., 2016; Howard‐Varona et al., 2017; Williamson et al., 2002). Prophages thus provide an additional route by which environmental effects propagate rapidly through microbial communities and beyond.

Importantly, the effects of prophages can be less direct when they are mediated via evolutionary processes. Even then, they can result in cascading consequences when pleiotropic changes are involved. These kind of indirect effects are more subtle and challenging to identify, but their consequences on global ecosystems should not be ignored. For example, the presence of a prophage in a bacterium could select for some degree of phage resistance minimizing the impact of a switch to lytic cycle on the bacterium population. The acquisition of phage resistance often involves changes in the properties of the bacterial membrane which can in turn affect other traits, such as bacterial fitness and virulence (Cota et al., 2015; Flyg et al., 1980; Zhang et al., 2014). Temperature is an environmental factor of particular interest for this type of evolutionary dynamics, as it can both trigger lytic life cycles and affect the fitness cost of bacterial resistance to lytic phages (Quance & Travisano, 2009). Given how common prophages are in natural populations, it is urgent to better understand how global temperature changes and prophage‐bacterium co‐evolutionary dynamics can act together to affect higher‐level processes. This requires prophage‐bacterium experimental systems where evolutionary responses to environmental changes can be documented at the level of the prophage and of the bacterium, and where the cascading effects on other biological levels can be characterized simultaneously. In this study, we comprehensively investigated such a system and report evidence confirming that environmentally inducible prophages affect other biological levels not only in direct ways but also indirectly via evolutionary processes, thus propagating selection pressures due to environmental changes and resulting in the evolution of virulence of their bacterial hosts.

**FIGURE 1:**
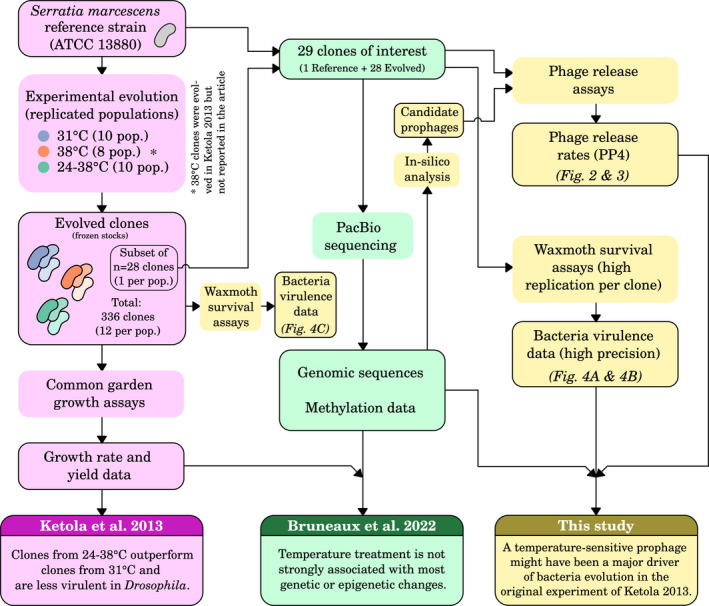
Overview of the relationships between our study and previous studies based on the same evolution experiment (Bruneaux et al., 2022; Ketola et al., 2013). Boxes are colour‐coded based on which study each item originates from.

We used clones from an evolution experiment where the bacterium *Serratia marcescens* had been exposed to three different temperature regimes (Ketola et al., 2013; Figure 1). *S. marcescens* is an opportunistic pathogen found in water and soil that can be virulent in plants, insects and vertebrates and is responsible for nosocomial infections in humans (Flyg et al., 1980; Grimont & Grimont, 2006). As such it is exposed to various temperatures in nature, both inside and outside plant or animal hosts. Its optimal growth temperature is close to 31°C in the laboratory (Ketola et al., 2013; Figure S1) During the evolution experiment, replicated populations were left to evolve either at constant 31°C, under a daily fluctuating regime (24–38°C), or at a constant elevated temperature of 38°C (Ketola et al., 2013; Figure 1). It was shown that clones evolved under daily fluctuating temperatures had some fitness advantages compared with clones evolved at 31°C, but that their virulence in a fly host was reduced. In a follow‐up study of the genetic and epigenetic basis of adaptation to different temperatures in *S. marcescens* where we sequenced a subset of those evolved clones, a few rare sequencing reads suggested the possible existence of excised prophage DNA (Bruneaux et al., 2022; Figure 1). This experimental material thus offered a great opportunity to investigate in detail whether temperature‐sensitive prophages could play a role in driving some of the observed evolutionary changes in *S. marcescens* and, given the pathogenic character of this bacterium in insects, to determine whether coincidental changes in bacterium virulence took place (Zhang et al., 2014).

Here, we first confirmed that the genome of the original strain used in the evolution experiment harboured several prophages, and we demonstrated that one of them was released as phage particles in a temperature‐dependent manner. We then hypothesized that a temperature‐susceptible prophage was likely to have consequences on bacterial fitness and to have exerted some selection pressure during the evolution experiment, and we checked whether the evolved strains exhibited evolutionary changes in their phage release rates. We expected that such changes would be pleiotropic and, notably, could have rippling effects on bacterial virulence in a model insect host. This hypothesis was based on the well‐demonstrated links between resistance to phage and alteration of the bacterial cell wall (Labrie et al., 2010), which is also involved in bacterial pathogenicity (Cota et al., 2015; Flyg et al., 1980), and on the fact that induced cell lysis can also increase bacterial virulence by facilitating the release of endotoxins (Ramachandran, 2014). Finally, we used the sequencing data available for those evolved clones to try to link any evolutionary changes observed above with candidate molecular mechanisms based on the genetic and epigenetic changes taking place during the evolution experiment (Bruneaux et al., 2022; Figure 1). All in all, by shedding light on how temperature‐sensitive bacteriophages can affect the evolution of their hosts and indirectly impact other biological levels, our results strengthen the understanding of the feedback loops between viruses, ecosystems and environmental changes.

## 2 MATERIALS AND METHODS

### 2.1 Origin of the strains used in this study

Strains originated from an earlier evolution experiment (Figure 1; Ketola et al., 2013). To initiate the experiment, a freshly isolated *Serratia marcescens* ancestor derived from the ATCC 13880 reference strain was grown overnight at 31°C in low‐nutrient medium (SPL 1%, hay extract) and spread to 30 replicate populations (10 populations per treatment). The 400 μl populations were placed under constant 31°C, constant 38°C, or daily fluctuating (24–38°C) thermal treatments, with daily renewal of low‐nutrient medium. After evolving for 30 days, 12 clones were isolated from each population by dilution plating on LB agar plates. Clones were grown overnight in low‐nutrient medium and frozen to 100‐well Bioscreen plates (1:1 with 80% glycerol) in randomized order.

### 2.2 DNA extraction, sequencing and genome annotation

Details for DNA extraction, PacBio SMRT sequencing and genome annotation are reported in a previous study (Figure 1; Bruneaux et al., 2022). One frozen clone per replicate population was randomly chosen for sequencing, as well as the original reference strain. Frozen stocks were grown in overnight precultures followed by 24h growth in 150 ml of SPL 1%. Since two populations from the constant 38°C treatment were lost during the experimental evolution, we sequenced 10 clones from constant 31°C, 10 clones from fluctuating 24–38°C, and eight clones from constant 38°C (Figure S2). Bacterial DNA was extracted from pelleted cells and sequenced on a PacBio RS II sequencing platform using P6‐C4 chemistry, with two single‐molecule real‐time sequencing (SMRT) cells run per DNA sample. Standard PacBio analysis protocols were used to assemble genomes and to estimate methylation fractions for adenine bases. Genome annotation was performed using the NCBI Prokaryotic Genome Annotation Pipeline (PGAP; Tatusova et al., 2016).

### 2.3 Identification of prophages and quantification of release rates

#### 2.3.1 Detection of prophage candidates

We used the PHASTER prediction tool (Arndt et al., 2016) (https://phaster.ca/) to detect the presence of prophage candidates in the genome of the *Serratia marcescens* reference strain we sequenced. The submission to the PHASTER server was made on 2019‐04‐21 and five putative prophages were detected (Table S1).

#### 2.3.2 Phage particle count using qPCR

We developed a method based on qPCR to quantify the amount of extracellular phage particles in liquid cultures of *S. marcescens*. The detailed method and the associated statistical analysis are fully described in the Supplementary Methods. We used five specific primer pairs targetting each of the candidate prophage regions and one additional primer pair targetting a chromosomal, non‐prophage‐related bacterial gene (Table S2) to quantify the amount of prophage DNA copies relative to the amount of bacterial genome copies present in a sample using qPCR. In essence, an excess of prophage DNA copies would indicate that phage genomes were replicated outside the bacterial chromosome. To quantify released phage particles in particular, and given low expected induction rates and the logarithmic scale of the uncertainty of qPCR estimates, our approach to reliably quantify the minute excess of extracellular prophage DNA was the following (Figure S3): (i) split a bacterial culture sample to be analysed into one raw sample and one supernatant sample obtained after gentle centrifugation to pellet bacteria cells, (ii) process both samples by DNase to digest DNA fragments which were not protected inside a bacterial cell nor inside a phage particle, (iii) inactivate DNase and release DNA from cells and phage particles by heating the samples at 95°C and (iv) quantify the amount of bacterial genome copies and of prophage DNA copies in both samples with qPCR. The supernatant sample is expected to be impoverished in bacterial cells while phage particles can remain in suspension, and thus, the proportion of prophage DNA copies which were not contained in bacteria cells in the culture (i.e. which were presumably in phage particles) can be estimated from the differential decrease in qPCR estimates for prophage DNA copies and for bacterial genome copies between the raw and supernatant samples. A Bayesian model was implemented to estimate phage‐to‐bacteria ratios based on qPCR results.

#### 2.3.3 Quantification of phage release rates in evolved strains

Using the approach outlined above, release rates of the five candidate prophages (i.e. all prophage candidates identified by PHASTER, irrespective of the prediction for prophage completeness) were tested under five temperature assay conditions in the 28 evolved clones selected for sequencing and in the original reference strain. The assays lasted 2 days and were made in SPL 1% under one of the following treatments: 31–31°C, 24–24°C, 38–38°C, 24–38°C and 38–24°C, where the temperatures are the temperatures on the first and second day, respectively, with a transfer to fresh medium between them (Figure S3). The details of the Bayesian model used to estimate the effect of evolutionary treatment and of assay temperature are given in Supplementary Methods.

### 2.4 Comparative genomics of prophage PP4

#### 2.4.1 Annotation of PP4

Since the candidate prophage PP4 was the only one for which induction was detected in our assays, we focussed our annotation and comparative analysis efforts on this prophage. The PHASTER output for PP4 contained a set of contiguous CDS in the *S. marcescens* genome which were related to phage functions. It also provided putative locations of attL/attR attachment sequences but those were located between prophage CDS instead of being at their periphery. We searched for better attL/attR candidates by looking for the longest repeated motif located 3000 bp upstream and downstream of the proposed prophage CDS. We found better candidates for attL/attR which encompassed all the PP4 CDS: the corresponding motif was 20‐bp long, compared with the 12‐bp long motif found by PHASTER. We defined PP4 as the sequence encompassed by those attL/attR sequences.

Annotation of PP4 CDS was manually curated by merging the annotation obtained for the *S. marcescens* genome (as described above and in Bruneaux et al., 2022) and the annotation provided by PHASTER. A last attempt to identify the CDS for which the ‘hypothetical protein’ status remained at this stage was performed by blasting their predicted protein sequences against the nr database using NCBI blastx server and its default settings, but excluding the *Serratia* taxid (blast run on 2020‐04‐10).

#### 2.4.2 Identification of phages and prophages related to PP4

To identify phages related to PP4, we retrieved all the phage genomes available from RefSeq Nucleotides using the query Viruses[Organism] AND srcdb_refseq[PROP] NOT wgs[PROP] NOT cellular organisms[ORGN] NOT AC_000001:AC_999999[PACC] AND (“vhost bacteria”[Filter]). The database was searched on 2020‐04‐01 and returned 2522 phage genomes. The nucleotide sequence of PP4 was compared with those phage genomes using a local blastn search (task blastn). The three phage genomes giving the total best scores were temperate phages: P88 which infects *Escherichia coli* (KP063541) (Chen et al., 2017) and SEN4 and SEN5 which infect *Salmonella enterica* (KT630645 and KT630646) (Mikalová et al., 2017).

To identify bacterial genomes containing prophages related to PP4, we ran a blastn search of the nucleotide sequence of PP4 against the NCBI nt database (excluding *Serratia* taxid) on 2020‐04‐02. The bacteria genomes giving the best total scores were *Klebsiella oxytoca* KONIH1 (CP008788.1) and *K. oxytoca* KONIH4 (CP026269.1), which gave very similar scores. We selected KONIH1 for further analyses. To determine the precise locations of PP4‐related prophages in the chromosome of KONIH1, we ran a local tblastx search of its sequence against PP4 and produced a dot plot using the tblastx matches. We visually identified three segments showing a consistent matching structure with PP4. For each segment, we determined the best attL/attR candidates using the same approach used when refining the attL/attR sites for PP4 as described above. This enabled us to extract three prophages (KONIH1/1–3). For each of these prophages, we extracted the CDS contained between their attL/attR based on their GenBank record. Finally, we also searched the genome of the *Serratia* strain we used in our study for other prophages similar to PP4, using the same local tblastx approach as described for KONIH1. The dot plot showed that PP7 was related to PP4, and we included PP7 in our final comparisons.

Once we extracted the nucleotide sequences for phages P88, SEN4, KSP20 (two fragments available: AB452988.1 and AB452989.1) and for prophages KONIH1/1–3 and PP7, we identified the matches between their predicted proteins and those from PP4 by running a final local tblastx search of each of those sequences against the PP4 sequence.

### 2.5 Detection of phage particles by other methods

#### 2.5.1 Transmission electron microscopy

Two precultures of the *S. marcescens* reference strain were grown overnight at 31°C in 10 ml of SPL 1% and used to inoculate two volumes of 100 ml of SPL 1%. After 10 h, the 100 ml cultures were each added to 300 ml of fresh medium and culture continued for 17 h at 31°C. Cells from the two 400 ml batches were pelleted separately (20 min at 10900 *g* with Sorvall RC‐6+ centrifuge and F12‐6x500 rotor), and their supernatants were filtered with 0.45 μm filters. The filtered supernatant from one 400 ml batch was then pelleted (1.5 h at 45000 *g* at 4°C with Beckman Coulter L‐90 K centrifuge and 45 Ti rotor), and supernatant was discarded. The filtered supernatant from the second 400 ml batch was added, and a second centrifugation (1.5 h at 45000 *g*) was applied. Pellets were resuspended in 1 ml of 0.1 m ammonium acetate and combined. After a last round of centrifugation with the same settings, the final pellet was resuspended in a total of 100 μl of 0.2 m Tris–HCl and stored at 4°C until staining for TEM. For staining, 1:10 dilutions of pellet (10 μl) were placed for 1 min on glow discharge‐treated copper grids. Staining was done with 1% phosphotungstic acid (PTA) for 2 min. Imaging was performed with JEOL JEM‐1400HC at 80 kV. The resuspended pellet was also used in qPCRs similar to the ones used to quantify phage release rates, using all the candidate prophage‐specific primers, to confirm the identity of the phage particles observed in TEM.

#### 2.5.2 Plaque assays

The *S. marcescens* reference strain was grown in liquid medium at room temperature and at 37°C. Supernatants and cells were prepared from both growth conditions. Bacteria (100 μl) were spread on top of 1% agar plates, and the double‐layer agar method was also employed by mixing 300 μl with 3 ml of 0.7% soft agar and poured on top of the solid agar. Supernatants (10 μl drops) were applied on plate cultures of the *S. marcescens* reference strain itself, of two freshwater *Serratia* strains, and of Db10 and Db11 *Serratia* strains grown at room temperature and at 37°C. No plaque was observed for any combination of growth condition of the reference strain with any receiving plate. Additionally, a separate experiment where mitomycin C was added (1 μg ml^−1^) to liquid cultures of the *S. marcescens* reference strain either at room temperature or at 37°C did not result in any visible clearing.

### 2.6 Bacterial virulence assays in an insect model

We estimated the virulence of our *S. marcescens* strains by measuring the longevity of waxmoth larvae (*Galleria mellonella*) injected with 5 μl of bacterial culture. *G. mellonella* is a good model for bacterial virulence as it has been shown that virulence in this host is also a good proxy for virulence in vertebrate hosts such as mouse or fish (Djainal et al., 2020; Jander et al., 2000). We grew bacterial cultures of evolved strains overnight at 31°C in Bioscreen wells in 400 μl of SPL 1% inoculated with the strains frozen stocks using a cryoreplicator. The reference strain was similarly grown overnight at 31°C in 8 ml of SPL 1% in a loose‐capped 15‐ml tube inoculated from a frozen sample. On injection day, culture optical densities (OD) were measured, and each larva was injected with 5 μl of a single culture in the haemocoel with a Hamilton syringe. The bacterial densities for most cultures were approximatively 0.6 to 4.5 × 10^8^ CFU/mL, based on OD values. For each strain, 20 larvae were injected simultaneously; ten of those were then incubated at 24°C while the other ten were incubated at 31°C. Larval survival was monitored at 1–3 h intervals by checking for larva movements, and time of death was recorded as the inspection time when a larva was found unresponsive. Additionally, for each incubation temperature, ten larvae were injected with sterile medium and ten with sterile water as controls. This set‐up was replicated four times, resulting in a total of 80 infected larvae per strain (40 to incubation at 24°C and 40 to incubation at 31°C). Some larvae from the first replication block were discarded due to a technical problem, leaving three replication blocks instead of four for some strains.

We analysed the larval survival data using a Cox proportional hazards model, where replication block, larval body mass, culture optical density, strain identity, incubation temperature and the interaction between strain identity and incubation temperature were included as fixed effects. In this type of model, the hazard function describes the instantaneous rate of death at a given time *t* for an individual still alive at *t*. The model included the effect of strain evolutionary treatment on their virulence, using a hierarchical Bayesian approach in JAGS 4.1.0 (Plummer et al., 2003; Su & Yajima, 2015) with the R2jags package. The proportional hazards were implemented as described by Clayton, 1991 (Clayton, 1991) based on code from the OpenBUGS Examples (The OpenBUGS Project). The details of the model are presented in the Supplementary Methods.

### 2.7 Analysis of genetic and epigenetic variation

We used the genomic and methylation data for the 28 evolved clones of interest and the reference strain available from Bruneaux et al., 2022 (Figure 1). As reported in this previous study, each sequenced clone was indeed genetically clonal, and loci variable across clones were identified from an alignment of the 29 sequenced genomes produced by Mugsy (Angiuoli & Salzberg, 2011; Table S3). To investigate the association between genetic variation and phenotypic traits (phage release and virulence in waxmoth larvae), we ran Wilcoxon rank sum tests for all combinations of genetic variants and traits, using only genetic variants present in at least two strains. *p*‐values were corrected for multiple testing using the false‐discovery rate method (Benjamini & Hochberg, 1995).

Epigenetic data consisted of the methylation fraction for adenosine bases in all GATC motifs present in the reference strain genome (38,150 GATC palindromes were present in the reference strain genome, corresponding to 76,300 adenosine bases for which methylation fraction values were analysed). Since the vast majority of the adenosines present in GATC motifs were fully methylated in all sequenced strains, we analysed the subset of GATC motifs which exhibited low methylation level in at least one strain as described in Bruneaux et al., 2022: 483 palindromes corresponding to 966 adenosines were used for association analysis with phenotypes (1.2% of all the adenosines in GATC motifs). The significance of the association between each of these 966 epiloci and a given phenotypic trait was calculated as the *p*‐value for Spearman's *ρ* correlation coefficient between the phenotype values and the m6A methylation fractions for the 29 sequenced strains. We used Spearman's *ρ* (i.e. rank correlation) to avoid excessive leverage from extreme phenotypic values.

To gain insights into potential functional roles of those epiloci, they were associated with annotated genes. A gene was assigned to an epilocus if the adenosine base was located within the gene coding region, or less than 500 base pairs upstream of the initiation codon to cover potential regulatory regions of the gene. Several gene set approaches were then tested to try to detect biological functions or pathways related to the epiloci associated with phenotypic traits. We used gene‐ontology enrichment tests as implemented in the TopGO R package and KEGG pathway analysis with Wilcoxon rank sum statistics to compare gene sets, but mostly, only very general biological functions were detected with those approaches, such as amino acid or carbon metabolism, nutrient transport and translation (data not shown). Since those approaches are targetting the detection of changes affecting a given biological function or pathway on average but are not efficient to detect single genes which might affect phenotype, we decided to generate lists of top candidate genes associated with each phenotypic trait (using uncorrected *p*‐value < 0.005 for Spearman's *ρ* correlation as the threshold) and to manually curate those genes. Manual curation entailed a literature search to provide a brief description of the function of the gene product in bacterial species and to flag genes potentially involved in chosen categories of interest: regulation of transcription, nutrient transport, excretion, cell wall structure, virulence and a larger last category embracing motility, biofilm formation, adherence and quorum sensing.

### 2.8 Statistical analyses

All statistical analyses were performed with R version 4.1.1 (R Core Team, 2021). Estimates from Bayesian models are reported in the text as posterior means with 95% credible intervals in square brackets. All scripts and data needed to reproduce the analyses are available on the Dryad repository (https://doi.org/10.5061/dryad.f4qrfj701).

## 3 RESULTS

### 3.1 Identification of a temperature‐sensitive prophage in *S. marcescens* genome

**FIGURE 2:**
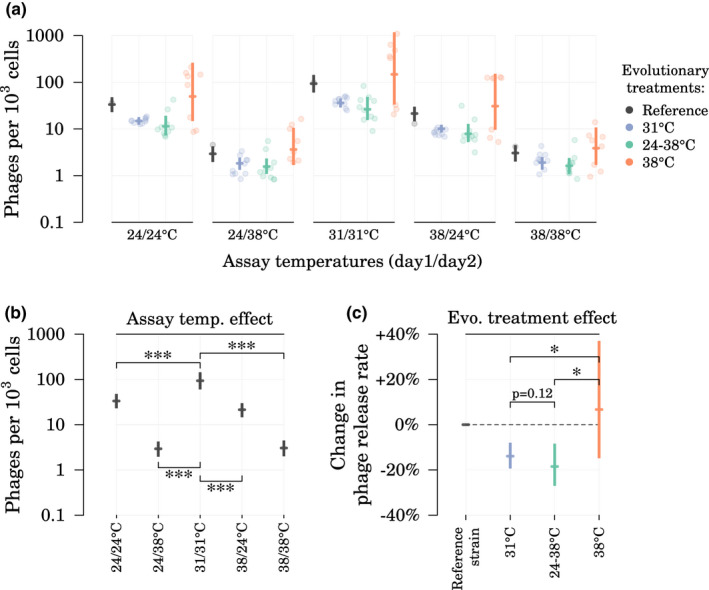
Effect of evolutionary treatment and assay temperatures on the release rates of prophage PP4. Assays lasted 2 days and assay temperatures are given as day1/day2. (a) Posteriors of the model‐estimated mean for each treatment/assay combination. Points are estimated phage release rates for each of the 29 sequenced clones. Full posterior estimates for individual strains are shown in Figure S5. (b) Estimates of the assay temperature effects and (c) estimates of the evolutionary treatment effects, with the reference strain used as a reference point. Posteriors are shown as median and 95% credible interval. One‐sided Bayesian p‐values for pairwise comparisons denoted by * (*p* < 0.05) and *** (*p* < 0.001).

Since prophages are ubiquitous in bacterial genomes, we expected to detect some in *S. marcescens* genome; we also expected that some could be temperature‐susceptible since *S. marcescens* is an environmental pathogen. In‐silico analysis of the genome of the reference strain (ATCC 13880) with PHASTER (Arndt et al., 2016) predicted the existence of five prophage candidates, with two of them incomplete based on PHASTER completeness score (Table S1). Using our qPCR‐based method to determine phage release rates in liquid cultures under a variety of temperature assays and prophage‐specific primers targetting all five prophage candidates, we detected extracellular phage particles only for prophage PP4 across the sequenced clones (Figure 2a). The release of PP4 particles by *S. marcescens* was temperature‐sensitive: on average, the lowest rates were observed at 38°C (3 [2–4] phages per 10^3^ cells) and the highest rates at 24°C (33 [23–48] phages per 10^3^ cells) and 31°C (94 [58–145] phages per 10^3^ cells) (Figure 2b, estimates for assays at 38/38°C, 24/24°C, and 31/31°C). From a phage genomics point of view, a comparative analysis showed that PP4 had very high sequence similarity with the *S. marcescens* phage KSP20 previously isolated from aquatic environment (Matsushita et al., 2009) and with P2‐like temperate phages and prophages from Enterobacterales (Figure S4). We did not find any known virulence factors among the identified proteins of PP4; however, 12 of its 44 predicted proteins remained unannotated (Figure S4).

**FIGURE 3:**
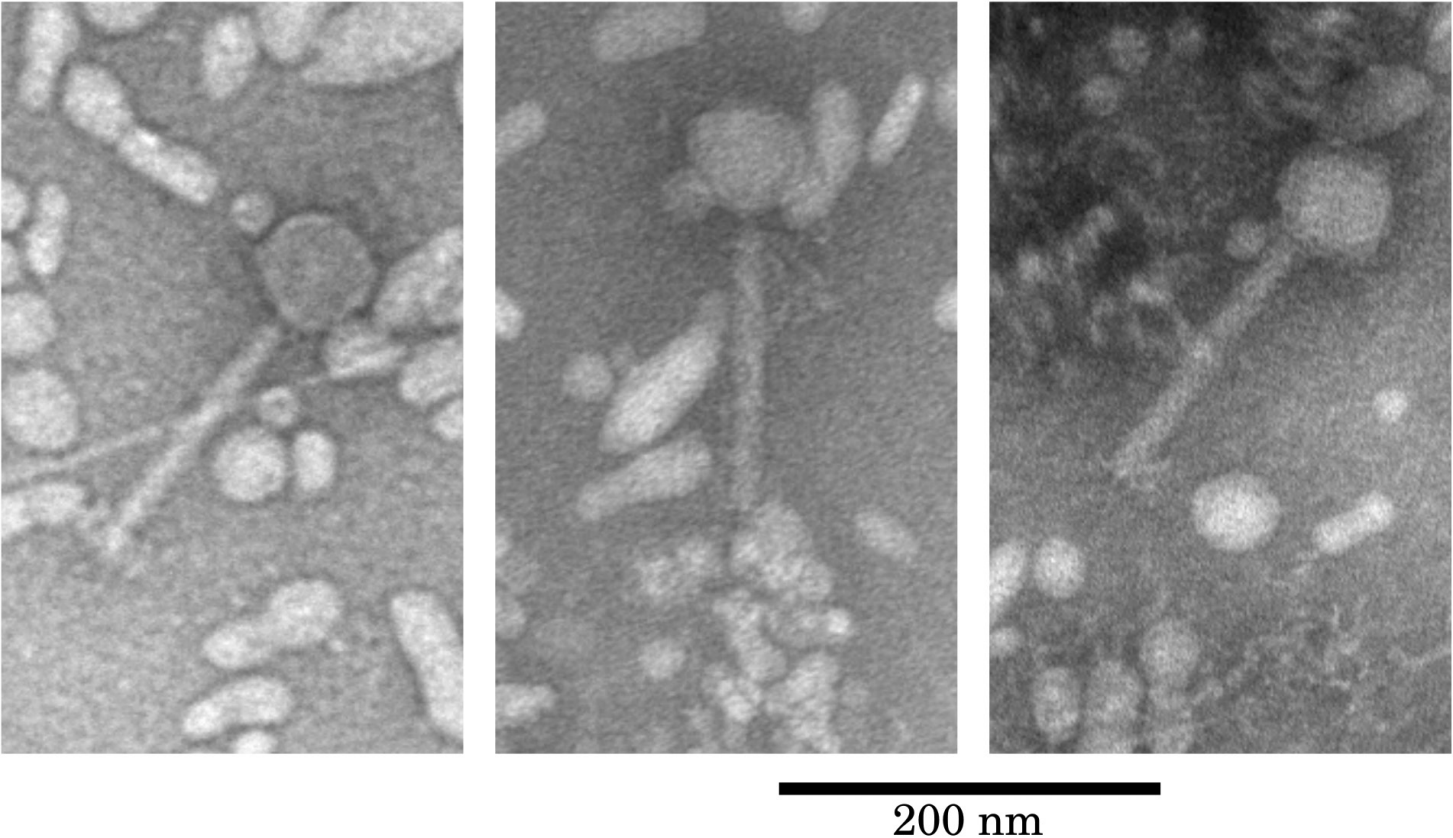
Putative PP4 phage particles observed in transmission electron microscopy (TEM). Negative staining with phosphotungstic acid (PTA) was used. All panels are shown to the same scale.

We performed two additional experiments using independent approaches to confirm the presence of PP4 phage particles in our liquid cultures. The first approach was to search for phage particles from pellets prepared from the supernatant of a reference strain culture grown at 31**°**C using transmission electron microscopy (TEM). Particles of shape and size compatible with a P2‐like phage were successfully observed, albeit in low frequency (Figure 3). The phage identity was confirmed by qPCR using PP4‐specific primers on the TEM pellets. The second approach consisted in plaque assays with supernatants from reference strain cultures spread onto lawn cultures of several candidate strains, including the reference strain, but no plaque was observed in any of the conditions tested.

### 3.2 Evolutionary changes in phage release rates

Assuming that the temperature‐dependent release of phage particles decreased bacterial fitness in the laboratory conditions used (i.e single‐strain cultures), we hypothesized that evolutionary changes in phage release rates would occur during the evolution experiment under different temperature regimes. Using 2‐day thermal assays to measure phage release rates (Figure 2a), we evaluated both the effect of mean temperature and of temperature fluctuations on phage release rates in the evolved clones. We found an evolutionary adjustment of prophage release rates in clones evolved under cooler environments (evolutionary treatment effect, Figure 2c). The strains evolved at 31°C and at 24–38°C released 20% [1%–34%] and 25% [7%–41%] less phages, respectively, than strains evolved at 38°C. Mean patterns of PP4 release rates did not differ significantly between strains that had evolved at lower mean temperature (31°C vs. 24–38°C). Compared with the reference strain, these evolutionary changes corresponded to a significant decrease of phage release in strains evolved at 31°C or 24–38°C, while the high variability in phage release rates across strains evolved at 38°C resulted in no significant difference with the reference strain (Figure 2c). However, when looking in more detail at phage release rates per strain, five of eight strains evolved at 38°C exhibited significantly higher phage release rates than the reference strain when assayed at 24/24°C, 31/31°C, and 38/24°C (Figure S5). When comparing assays under different temperature regimes, the main driver of phage release was the temperature on the final day of the assay, rather than whether a temperature change was experienced during the assay (assay temperatures effect, Figure 2b). Ending an assay at 31°C resulted in about three times more phages than ending an assay at 24°C (ratio between release rates at 31/31°C and 24/24°C: 2.8 [2.2–3.6]), and ending an assay at 24°C resulted about ten times more phages than ending an assay at 38°C (ratio between release rates at 24/24°C and 38/38°C: 11.2 [7.6–16.6]).

Since both evolutionary treatment and assay temperature have an effect on bacteria maximum growth rates (Figure S1; Ketola et al., 2013), their overall effect on phage release rate could be mediated to some extent by their effect on bacteria growth rate which could, in turn, affect phage release. We checked for this by updating our statistical model to take into account bacteria maximum growth rates at the temperature of the final days of the assays. This supplementary analysis showed that the effect of evolutionary treatment and of assay temperature on phage release was almost entirely independent from their effect on bacteria maximum growth rate (Figure S6; see Supplementary Results for details).

### 3.3 Evolutionary changes in bacterium virulence

**FIGURE 4:**
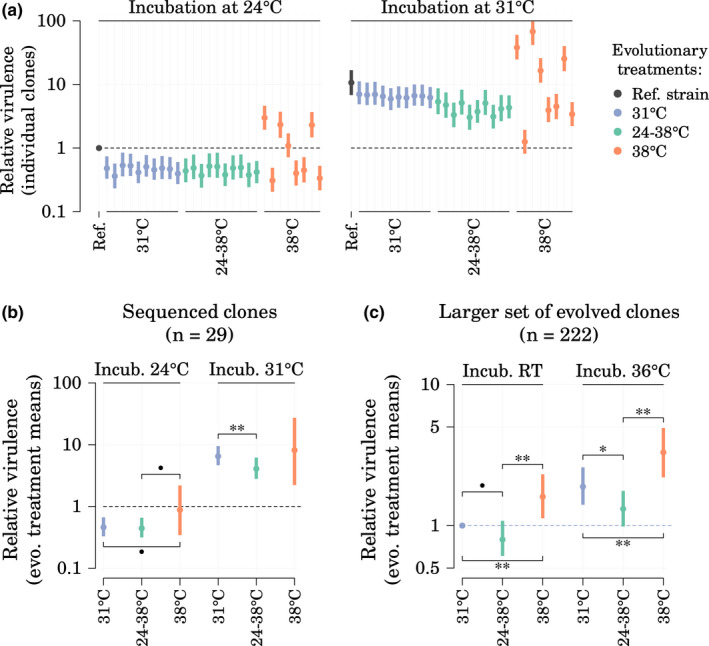
Effect of evolutionary treatment on strains virulence in waxmoth larvae at two incubation temperatures. (a) Relative virulence of individual sequenced clones, measured as relative hazards estimated from a Bayesian implementation of a Cox proportional‐hazards model. All virulence estimates are relative to the virulence of the reference strain in incubation at 24°C (denoted by a broken horizontal line) and are corrected for the effects of injection batch, larval body mass and optical density of injected cultures. (b) Mean relative virulence per evolutionary treatment and per incubation temperature as estimated by the model (exp(*μ*_evo_)) (*n* = 29 sequenced clones). (c) Confirmatory results from a similar virulence experiment utilizing more bacterial clones from the same original evolution experiment (*n* = 222 clones, incubation at room temperature (RT) and at 36°C, virulence relative to the average virulence of the clones evolved at 31°C when incubated at room temperature). For each model parameter, 95% credible interval and median of the posterior are shown.

Changes in phage‐bacterium interactions often have pleiotropic effects affecting bacterial virulence in particular. We predicted that the observed evolutionary changes in phage release rates in *S. marcescens* could also affect *S. marcescens* virulence in some of its hosts. We estimated the virulence of the experimentally evolved strains by measuring the survival time of waxmoth larvae (*Galleria mellonella*) infected by an injection of 5 μl of bacterial cultures and placed into two assay environments: 24°C and 31°C (Figure S7). We did not use 38°C since some earlier experiments showed that *G. mellonella* inoculated with *S. marcescens* died rapidly at this temperature, making collection of accurate survival times challenging. We estimated the relative virulence of sequenced strains from larvae survival data using a Cox proportional hazards mixed model which controlled for both larva body mass and initial density of bacterial cultures and which allowed for different variances across evolutionary treatments (Figure 4a). Those estimates revealed that the average virulence of clones evolved at high temperature (38°C) tended to be higher than for clones evolved at lower mean temperature when larvae were incubated at 24°C (relative virulence at 24°C for strains evolved at 38°C, 24–38°C, and 31°C, respectively: 0.99 [0.34–2.19], 0.46 [0.32–0.65], and 0.47 [0.33–0.65]; comparison 38°C versus 24–38°C, one‐sided Bayesian *p*‐value = 0.057; 38°C versus 31°C, *p* = 0.066; Figure 4b). However, when larvae were incubated at 31°C, these differences disappeared while clones evolved at 31°C were more virulent than those evolved at 24–38°C (*p* < 0.01) (relative virulence at 31°C for strains evolved at 38°C, 24–38°C, and 31°C, respectively: 9.68 [2.09–25.59], 4.22 [2.87–6.19], and 6.64 [4.89–9.42]). This was consistent with previous results from Ketola et al. (2013) showing that the clones evolved at 31°C were more virulent than clones evolved at 24–38°C in a *Drosophila* model. Like for the phage release results above, we also checked whether the effects of evolutionary treatment and incubation temperature on virulence could be mediated by an intermediate effect on bacterial growth rate which could, in turn, affect bacterial virulence. A supplementary analysis where we conditioned the model on bacterial growth rates produced very similar mean estimates for those effects, with slightly increased uncertainty, suggesting that those effects were mostly independent from growth rates (Figure S8).

**FIGURE 5:**
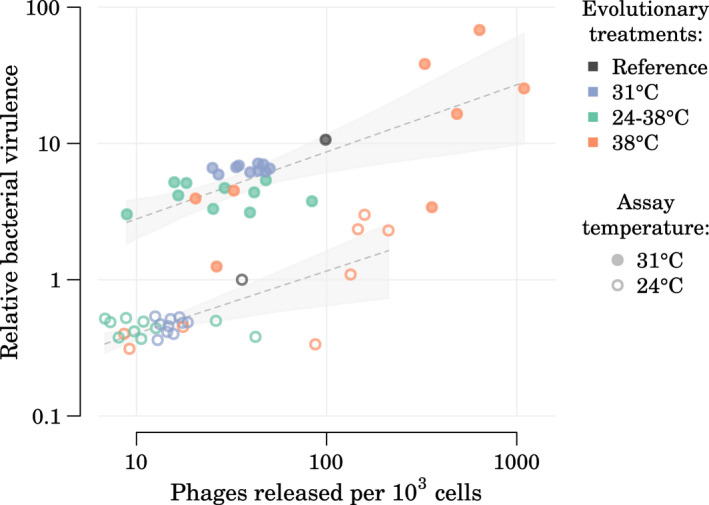
Relationship between relative bacterial virulence in waxmoth larvae and phage release rate for the sequenced evolved strains used in this study (*n* = 28) and the reference strain. Each data point represents one strain at a given assay temperature (24°C: data from virulence assay at 24°C and phage release assay at 24/24°C; 31°C: data from virulence assay at 31°C and phage release assay at 31/31°C). Bacterial virulence is relative to the reference strain in incubation at 24°C, similarly to Figure 4. Trend lines within each assay temperature are added for visual support only and are built using ordinary least squares regressions (95%‐enveloppes built using 500 bootstraps).

To confirm the virulence results obtained from the 29 sequenced clones, we also measured virulence in waxmoth larvae for a much larger pool of evolved clones from the same original evolution experiment (Figure 1) which confirmed that overall clones evolved at 38**°**C had indeed a higher virulence than the others when assayed at room temperature (*p* < 0.01 for comparisons of 38**°**C clones with both 24–38**°**C and 31**°**C clones, Figure 4c). Finally, we observed a moderate‐to‐strong positive correlation between strain virulence in waxmoth larvae and PP4 release rates (Figure 5; Spearman's *ρ* = 0.39, *p* = 0.039 between virulence at 24**°**C and phage release at 24/24°C; *ρ* = 0.58, *p* = 0.001 between virulence at 31**°**C and phage release at 31/31**°**C).

### 3.4 Association between genetic and epigenetic variation and phenotypic changes

**FIGURE 6:**
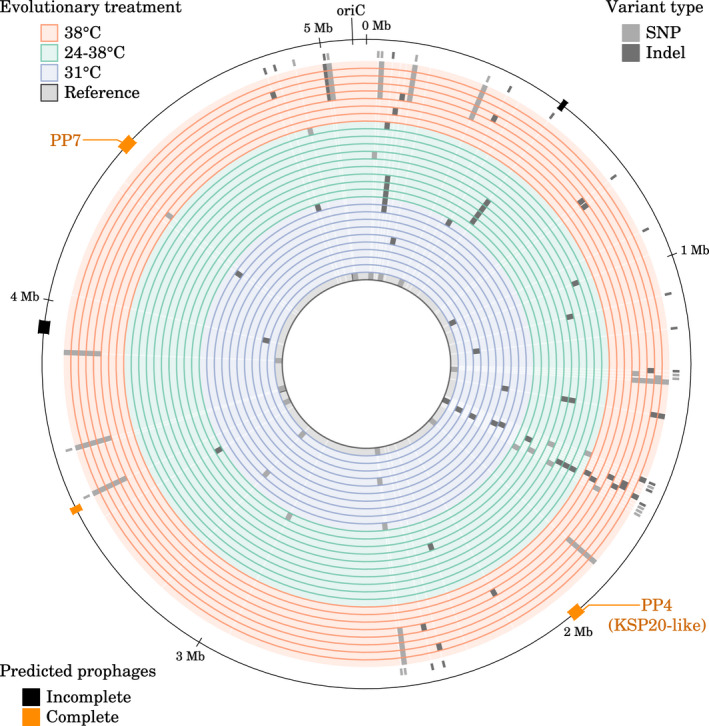
Alignment of the genomes from the 29 sequenced strains showing the variable genetic loci. Each circular track represents a sequenced genome, for which the evolutionary treatment is colour‐coded. Minor alleles for genetic variants are shown on the genome tracks in light grey (SNPs) and dark grey (indels). Ticks outside the last genome track indicate nonsynonymous variants (i.e. nonsynonymous SNPs and indels resulting in a frame shift). The outer line represents coordinates along the genome and the locations of the five predicted prophages. Prophages PP4 and PP7 are the prophages shown in Figure S4.

Given that evolutionary changes were observed both in phage release rates and in bacterial virulence, heritable changes responsible for those must have taken place. We took advantage of available sequencing data to explore the genetic and epigenetic changes which occurred during the evolution experiment and to investigate the candidate mechanisms explaining those phenotypic differences. We investigated the association between phage release and virulence traits and genetic variants present in at least two strains using Wilcoxon rank sum tests adjusted for false‐discovery rate (Benjamini & Hochberg, 1995); we also used the methylation data obtained from PacBio SMRT sequencing to test for association between phenotypic traits and adenosine methylation in GATC motifs (which are recognized by an adenine methyltransferase in *S. marcescens*; Ostendorf et al., 1999; Bruneaux et al., 2022). Based on a genome alignment, 52 variable genetic loci were identified among the sequenced clones, but none was located inside prophage PP4 sequence (Figure 6 and Table S3; Bruneaux et al., 2022). Remarkably, haplotype *a* which contained 11 linked loci was observed only in five of eight clones evolved at 38°C and in the reference strain but in none of the sequenced clones from the other evolutionary treatments (Figure 6; Table S3). As explained in Bruneaux et al. (2022), this indicates that the ancestor culture used to initiate the replicate populations at the beginning of the evolutionary experiment probably exhibited some standing genetic variation related to this haplotype, maybe due to cell aggregation when isolating the ancestor colony. This allowed for both standing genetic variation and de novo mutations to be subject to selection during the original evolution experiment.

**FIGURE 7:**
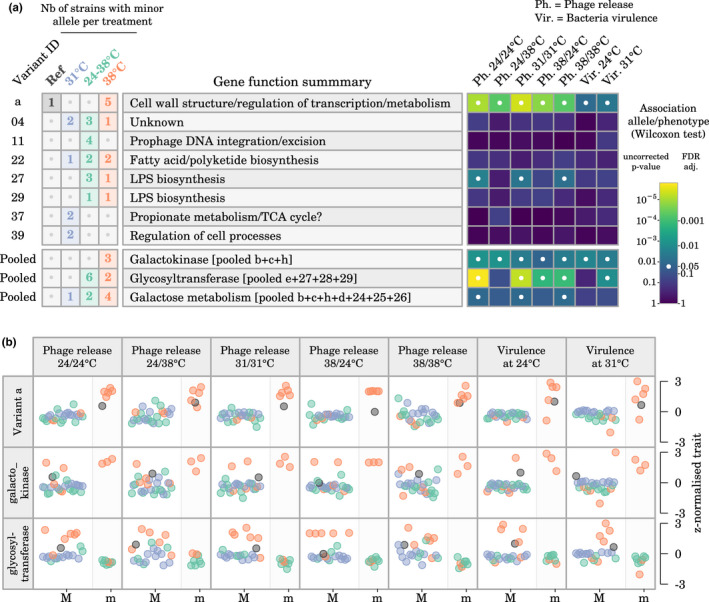
Association between phage release, virulence, and genetic variants observed in at least two sequenced strains. (a) Distribution of genetic variants across evolutionary treatments and association between alleles and phenotypes based on Wilcoxon rank sum tests. Variant IDs can be matched with those in Table S3 for details. (b) Visualization of the association between phenotypic values and major (M) and minor (m) alleles of variant *a* and of the “pooled” variants for galactokinase and glycosyltransferase. Colours correspond to the evolutionary treatment applied to each strain.

Several of the variable genetic loci associated with phage release and with bacterial virulence had a potential role in the biofilm structure and in the outer structure of the cellular envelope (e.g. genes involved in peptidoglycan and LPS biosynthesis; Figure 7 and Table S3). A striking pattern was the presence of three distinct mutations occurring in a single glycosyltransferase gene and close to the putative active site of the protein (mutations *28*, *29* and *30*; Figure S9; Table S3). These mutations were observed independently in three strains evolved at 24–38°C and in one strain evolved at 38°C (which had low phage release rates and low virulence compared with the other strains evolved at 38°C).

**FIGURE 8:**
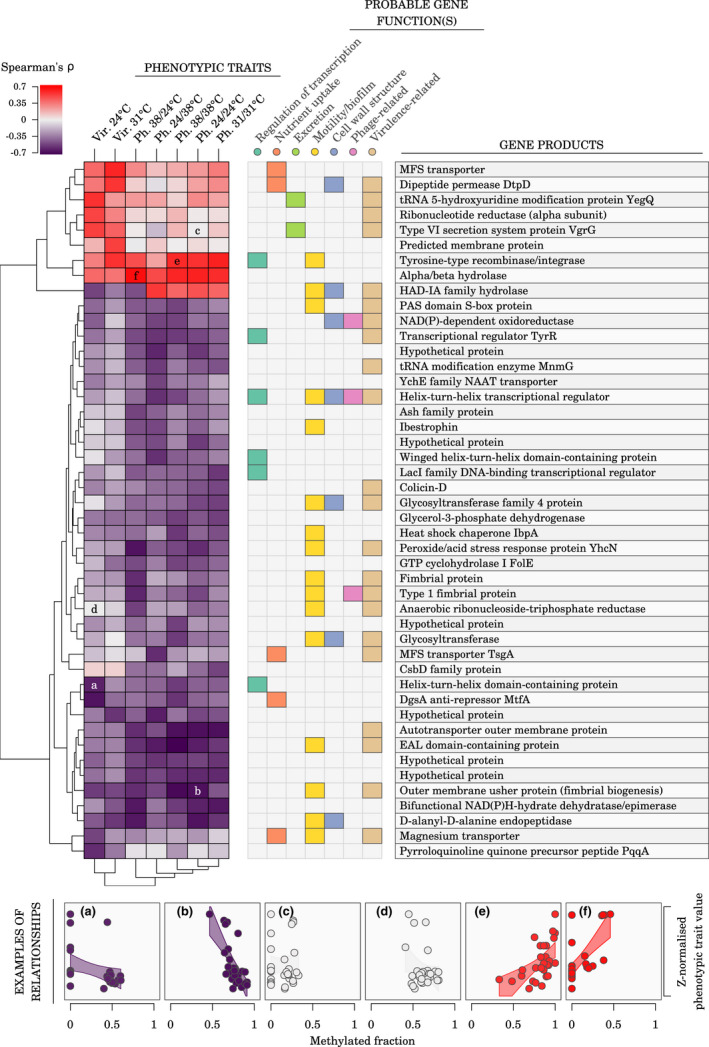
Association between phage release, virulence, and adenine methylation changes. The heatmap shows Spearman's *ρ* between methylated fractions of variable m6A epiloci (rows) and phenotypes (columns; Ph., phage release; Vir., bacteria virulence in waxmoth larvae). Overlapping or closest (≤500 bp) downstream genes were assigned to each m6A epiloci. Probable gene functions were assigned to each gene product based on a manual literature search. Visualization of the relationship between m6A methylated fractions and phenotypic trait values is shown for the heatmap cells marked with a letter. The m6A epiloci used in this figure were the variable m6A epiloci with a methylation fraction range ≥0.2 across sequenced samples and an uncorrected *p*‐value ≤0.005 for Spearman's *ρ* with at least one phenotypic trait.

Forty‐six genes were found associated with phenotypic changes via adenosine methylation changes, and we manually curated their functional annotation based on the literature (Figure 8). Many of those genes were involved in functional categories that are critical for pathogen virulence in other bacteria species, such as nutrient capture, excretion into the outer medium, biofilm formation, adherence and motility, all of which have a key role in successful colonization and invasion of the host tissues (Liu et al., 2017; Luo et al., 2017; Ren et al., 2018; Turner et al., 2009) (Figure 8). Additionally, several genes were involved in cell wall structure, notably in lipopolysaccharide biosynthesis.

## 4 DISCUSSION

### 4.1 *S. marcescens* carries a P2‐like, temperature‐inducible prophage

We used three independent methods (qPCR, TEM and plaque assays) to establish whether any of *S. marcescens* prophages could result in phage particles in our experiments. Taken together, our results confirmed that the PP4 prophage is induced in *S. marcescens* cells and released as extracellular phage particles at a low, temperature‐sensitive rate as detected by qPCR and confirmed by TEM, but suggest that the burst and/or infectivity rates of this phage are low enough that no clearing is observed in our plaque assays. Interestingly, the immunity repressor protein located immediately upstream of the PP4 integrase is of the short type (99 amino acid long) and thus most likely of the P2‐type rather than of the longer 186‐type (Christie & Calendar, 2016). Since phage 186 is inducible by the SOS response while phage P2 is not, this suggests that prophage PP4 might be noninducible by the SOS response like phage P2 (Christie & Calendar, 2016; Lamont et al., 1989).

Structural proteins were well conserved between PP4 and related sequences, as is common for P2‐like phages (Nilsson & Haggård‐Ljungquist, 2007), with the exception of tail fibre proteins (Figure S4). Tail fibre proteins are known to be involved in phage host specificity (Le et al., 2013; Scholl et al., 2001; Yehl et al., 2019) and P2‐like phages, while exhibiting strikingly similar structural genes even when infecting different species, have been suggested to coevolve with their bacterial hosts (Nilsson & Haggård‐Ljungquist, 2007)

### 4.2 Abiotic changes affect the evolution of prophage‐bacterium interaction

Given that *S. marcescens* carries a temperature‐inducible prophage, a comparison of phage release rates after bacterial evolution under different temperatures provides a compelling opportunity to explore how environmental changes can affect the evolution of prophage‐bacterium interactions. In particular, increased phage release rates are likely to have a negative effect on bacterial fitness in a monospecies population, assuming that increased release rates are at least partly due to increased induction and/or burst rates. The hypothesis that increased phage release co‐occurs with decreased bacterial fitness is supported by a moderate‐to‐strong negative correlation observed between phage release rate and bacterial yield based on the strain growth data from Ketola et al. (2013) (Spearman's *ρ* = −0.51, *p* = 0.006 at 24°C and *ρ* = −0.46, *p* = 0.015 at 38°C for *n* = 28 sequenced evolved strains; Figure S10). It is thus reasonable to expect that selection would favour counter‐adaptations in *S. marcescens* to reduce the fitness loss due to prophage induction and cell lysis in cooler environments (Canchaya et al., 2003), while evolution in warmer environments would release selective pressure against such fitness loss. This prediction was confirmed based on the phage release rates we measured for evolved clones: the evolution of lower phage release rates in clones from evolutionary treatments where prophage‐inducing temperatures occurred (i.e. treatments 31°C and 24–38°C) was consistent with a temperature‐dependent effect of PP4 on *S. marcescens* fitness.

Since bacterial growth rates are known to be affected by the evolutionary treatment in the original evolution experiment (Ketola et al., 2013), the effect of evolutionary treatment on phage release rates could potentially be mediated by the evolutionary changes in bacteria growth rates, which would in turn affect phage release rates. We investigated this possibility by including maximum growth rate values for the 28 strains of interest, taken from the original dataset from Ketola et al. (2013), into our statistical model (Figure S6; Supplementary Results). Conditioning on bacteria growth rates did not change notably the model estimates for the effect of evolutionary treatment and assay temperature, suggesting that the evolutionary changes in phage release rates are not mediated by the changes in growth rates but rather represent a separate evolutionary component. This is visible in particular when examining the growth rates for the five strains evolved at 38°C presenting the highest phage release rates in our dataset, and which did not differ markedly from the other strains in terms of growth rate (Figure S1).

### 4.3 Induced evolutionary changes have consequences for bacterium virulence

After confirming that evolutionary changes occurred in the interaction between prophage PP4 and its bacterial host, we explored how these environmentally triggered changes might cascade to another biological level: an insect host of the bacterium itself. Previous evolution experiments with the same bacterial species have shown that evolution in the presence of a protist predator or of a lytic phage affected *S. marcescens* virulence in an insect host (Friman et al., 2011; Mikonranta et al., 2012). In particular, a trade‐off between resistance to a lytic phage and bacterial virulence traits was demonstrated by Friman et al. (2011): the presence of the lytic phage prevented the evolution of more virulent *S. marcescens* clones at 37°C while virulence of bacteria evolving at the same temperature but without the lytic phage increased. Those studies support the coincidental evolution hypothesis, which suggests that selection pressures acting on an opportunistic pathogen in the external environment can affect the pathogen virulence in its host (Brown et al., 2012; Levin & Edén, 1990). In our experiment, we hypothesized that evolutionary changes related to phage release rates might also impact bacterial virulence. We observed substantial positive correlation between average strain virulence in waxmoth larvae and average PP4 release rates, which confirmed that phage release in liquid medium closely related to the bacteria virulence in the insect host and is in line with the coincidental evolution hypothesis. Interestingly, the original selection pressure in our case was abiotic (environmental temperature acting on phage release rates), contrary to the protist predator and the lytic phage used by Friman et al. (2011) and Mikonranta et al. (2012).

### 4.4 Both genetic and epigenetic variation can play a role in phenotypic changes

To explore the mechanistic basis of the evolutionary changes in prophage release rates and in bacterial virulence, and the potential connection between those two traits, we analysed the genomic and methylation data of the 28 evolved strains used in our study and of the reference strain pre‐existing the evolution experiment (Figure 1; Bruneaux et al., 2022). We found no known virulence factor among the annotated proteins of PP4, but some accessory proteins found in P2‐related phages are known to contribute to bacterial virulence, protection against other bacteriophages, or SOS prophage induction (Christie & Calendar, 2016). More generally, prophage‐encoded virulence factors are considered one of the benefits brought by prophages to their bacterial host explaining the maintenance of prophages in bacterial genomes (Fortier & Sekulovic, 2013; Koskella & Brockhurst, 2014). Another possible hypothetical connection between prophage induction and bacterial virulence could be that increased phage release rates would decrease bacterial survival in the host, hence leading to reduced virulence. However, this is not what we observed: despite a negative correlation between phage release rates and bacterial yields in laboratory cultures, strains with the highest phage release rates also had the highest virulence in the insect host. This suggests that if increased phage release had a direct effect on bacterial virulence, it would more likely be via the release of endotoxins upon cell lysis in the host since *S. marcescens* lysates are known to be cytotoxic on their own (Petersen & Tisa, 2012).

Given that none of the variable genetic loci we observed in our data set were located inside the prophage PP4 sequence, it can be reasonably expected that mutations elsewhere in the genome and/or epigenetic modifications should be responsible for evolutionary differences in phage release rates and bacterial virulence among the sequenced clones. Haplotype *a* seemed to be responsible for some of the high phenotypic variability observed in clones evolved at 38°C: five out of eight of those clones carried this haplotype and exhibited high phage release rates, and four of them also showed high virulence. Conversely, haplotype *a* was never observed in the clones evolved at 31°C and 24–38°C, which were more phenotypically homogeneous. More generally, the variable genetic loci we found associated with phage release rates and with bacterial virulence suggest that biofilm formation and outer cell wall structure can have a role in modulating those traits. In particular, the three distinct independent mutations in the glycosyltransferase gene we observed strongly support the hypothesis of a role of the outer cellular envelope in the evolutionary response against phages, with potential consequences on bacterial virulence. Similarly, among the genes for which variation in adenosine methylation was associated with changes in phage release rates or in bacterial virulence, several were related to lipopolysaccharide biosynthesis. All in all, these results indicate that the O antigen, which is typically involved both in cell recognition by phages and in bacteria virulence in their host (Chart et al., 1989; Li & Wang, 2012), could act as an important player in evolutionary trade‐offs between bacterial virulence and resistance to phage infection: improved immunity to phages at this level is likely to impair bacterial ability to infect. It has been noted previously that *S. marcescens* strains exhibit a large diversity of O antigens (Gaston & Pitt, 1989), that LPS biosynthesis genes are important for *S. marcescens* virulence (Kurz et al., 2003), and one particular study showed that *S. marcescens* cells grown at different temperatures had different LPS structures and phage affinities, with cells grown at 37°C having shorter O antigen and lower affinity for LPS‐specific phages than cells grown at 30°C (Poole & Braun, 1988). Those suggested links are consistent with earlier research: Flyg et al. (1980) showed that phage‐resistant *S. marcescens* mutants also had reduced virulence in *Drosophila* and Cota et al. (2015) demonstrated that an epigenetic mechanism altering the O‐antigen chain length in *Salmonella enterica* created a trade‐off between bacterial virulence and phage resistance.

Finally, a striking feature of the genetic and epigenetic changes observed in our study was that almost none of the associated genes was directly related with thermal selection pressure (only one heat‐shock chaperone was found, *IbpA*; Figure 8), even though temperature was the primary selective pressure in the evolution experiment. This is surprising, as the optimal growth temperature of *S. marcescens* is close to 31°C (Ketola et al., 2013). For instance, we did not find indication of changes related to other HSP or to DNAK genes in strains evolved under 38°C or 24–38°C compared with strains evolved under 31°C, even though they are known to be the target of selection in hot and fluctuating environments (Ketola et al., 2004; Sørensen et al., 2003, 2016). Such a weak direct effect of the experimentally manipulated factor underlines the fact that indirect selection due to phage release is likely to have overruled the direct effects of temperature. It is also consistent with the fact that the temperature treatment during evolution was not strongly asssociated with genetic or epigenetic changes (Bruneaux et al., 2022), but more associations are observed when we compare directly genetic and epigenetic data and phenotypic traits (which allows to capture the phenotypic variation between clones within an evolutionary treatment).

### 4.5 Areas of future research

The present study provides us with a global understanding of our experimental system and examines all the important aspects required to answer our original research question, but also uncovers new questions which deserve studies of their own. For example, what are the underlying mechanisms of evolutionary changes in phage release rates? Do they involve changes in prophage induction rates, in efficiency of phage particle assembly inside the cells, in burst rates or burst sizes, or in released phage adsorption onto the bacterial membrane? Do evolved strains with decreased phage release rates also gain some general phage immunity? Similarly, what are the mechanisms responsible for changes in bacterial virulence? Are membrane modifications important, or the increased release of endotoxin? In‐depth investigation of bacterial membrane structure and properties, such as interaction with phage particles and with the insect immune system, seems like the most promising avenue of research based on our analysis of genetic and epigenetic data. On a different aspect, a better characterization of the impact of phage release on bacterial fitness might shed light on the selection processes taking place during the evolution experiment itself. The relative fitness of evolved clones with high or low phage release rates could be estimated more precisely with head‐to‐head competition assays using pairs of evolved strains and methods such as high‐resolution melting assays (HRM) to quantify strains abundances after competition (Ashrafi et al., 2017). This would allow to calculate selection coefficients during experimental evolution, and to determine the temperature‐dependency of the cost of (pro)phage resistance if competitions were made at several temperatures.

Another axis for future research is to explore how generalisable our results are. We believe they are likely to be relevant to many environment‐prophage‐bacteria systems in nature given the key properties that led to our hypotheses, and that our results confirmed in our experimental system: (i) the presence of prophages in bacteria, (ii) the sensitivity to temperature of many prophages, (iii) the existence of a cost of phage release on bacterial fitness, with the resulting selection pressure leading to bacterial evolution and (iv) the pleiotropic effects of the ensuing molecular changes resulting in coincidental changes on other bacterial traits, such as virulence. The points (i), (iii) and (iv) seem frequent or easy to fulfil based on the literature, and the frequency of (ii) remains to be determined but can be extended to sensitivity to nutrient abundance, oxidative stress, or bacterium physiological status, so it is probably quite widespread when considering the sensitivity of prophage activation to environmental changes in general.

## 5 CONCLUSION

We showed that the opportunistic pathogen *S. marcescens* harboured a temperature‐sensitive prophage, and that phage release rates were susceptible to evolutionary changes. Consistent with the hypothesis that phage release decreased bacterial fitness (at least in laboratory conditions), we observed compensatory evolutionary trajectories where intrinsic release rates decreased in bacteria evolving under strongly release‐inducing temperatures (31°C and 24–38°C) while intrinsic release rates increased in some bacteria evolving under weakly release‐inducing temperature (38°C). Our experiments did not allow us to clearly identify which phage properties were affected by culture temperature (induction rate, burst size and/or capsid stability) and which molecular mechanisms were responsible for the evolution of different phage release rates in *S. marcescens*, but the genetic and epigenetic data were compatible with an important role of the structure of the outer cell wall. We also observed evolutionary changes in *S. marcescens* virulence, which were correlated with the changes in intrinsic phage release rates. Again, our genetic and epigenetic data are suggestive of mechanistic links probably involving outer cell wall structure, even if more research is needed to confirm the actual molecular mechanisms involved.

Our results provide an original instance of coincidental evolution where an abiotic environmental factor driving (pro)phage release rates in a bacterium had cascading effects on the evolution of bacterial virulence. Such occurrences of coincidental evolution can be hard to predict but are important to take into account to improve our understanding of the complex feedback loops between environment, viruses, microbes and ecosystems.

## AUTHOR CONTRIBUTIONS

TK, JAG and IK conceptualized the study. Experimental design for PacBio sequencing was made by TK and MB. DNA extraction for PacBio sequencing was done by RA. AMÖT identified prophage sequences. AMÖT, RA and MB designed the phage release assays and RA and MB performed the assay experiments. EL performed the electron microscopy experiments and the plaque assays and helped analyse PP4 sequence. CZ performed the virulence experiments of the sequenced clones in the insect host. RA, IK, MB, and TK performed virulence experiments for the larger pool of evolved clones. MKS assisted with laboratory experiments. MB and IK analysed the sequencing data. MB, IK and TK wrote the original draft with later edits and reviews by all co‐authors.

## CONFLICT OF INTEREST

The authors declare no competing financial interests.

## Supporting information


Appendix S1

## Data Availability

PacBio sequencing data (HDF5 files) were submitted to the European Nucleotide Archive's Sequence Read Archive (ENA‐SRA, https://www.ebi.ac.uk/ena, project PRJEB40306) and assembled genomes were submitted to NCBI's GenBank (biosamples SAMEA7301478 to SAMEA7301506). Genetic, epigenetic and phenotypic datasets used for analyses as well as R scripts needed to reproduce the results are available from the Dryad repository (https://doi.org/10.5061/dryad.f4qrfj701). The evolved clones of *Serratia marcescens* used in this study are available from the authors upon request. Benefits from this research accrue from the sharing of our data and results on public databases as described above.
