# Supplementary material for: The effect of a temperature‐sensitive prophage on the evolution of virulence in an opportunistic bacterial pathogen
Source: Mol Ecol. 2022 Sep 17;31(20):5402–18. doi: 10.1111/mec.16638 (PMC9826266; doi:10.1111/mec.16638)
Supplement: Supplementary file 1 — Appendix S1 [file MEC-31-5402-s001.pdf]

This document contains the supplementary materials for the article:

"The effect of a temperature-sensitive prophage on the evolution of virulence in an opportunistic bacterial pathogen"

by Matthieu Bruneaux, Roghaieh Ashrafi, Ilkka Kronholm, Elina Laanto, Anni-Maria Örmälä-Odegrip, Juan A. Galarza, Chen Zihan, Mruthyunjay Kubendran Sumathi, and Tarmo Ketola.

Contents

1 Supplementary Methods **3**
- 1.1 Quantification of phage release rates using qPCR . . . 3
  - 1.1.1 Culture conditions for the temperature assays . . . 3
  - 1.1.2 Sample processing and qPCR runs . . . 3
  - 1.1.3 Estimation of phage release rates and treatment effects using a Bayesian model . . . 4
- 1.2 Bayesian implementation of the Cox proportional hazards mixed model 8
- 1.3 Selection of m6A in non-fully methylated GATC motifs . . . 9

2 Supplementary Results **10**
- 2.1 Effect of bacterial growth rate on phage release rate . . . 10
  - 2.1.1 Statistical model update: adding bacteria growth rate effect . . 10
  - 2.1.2 Model outcome when conditioning on growth rate . . . 11
- 2.2 Effect of bacterial growth rate on bacterial virulence . . . 11
- 2.3 Association between genetic changes and phenotypic traits . . . 12
- 2.4 Association between epigenetic changes and phenotypic traits . . . 13

3 Supplementary tables **15**
- Table S1 In-silico detection of prophage sequences in *S. marcescens* genome . 15
- Table S2 Primers sequences (qPCR) . . . 15
- Table S3 Genetic mutations observed across the sequenced strains . . . 16

4 Supplementary figures **18**
- Fig. S1 Growth rates of evolved clones . . . 18
- Fig. S2 Setup of the original evolution experiment . . . 19
- Fig. S3 Overview of the experimental protocol of the prophage activation assays 20
- Fig. S4 Comparison of prophage PP4 with related (pro)phages . . . 21
- Fig. S5 Estimated release of phage PP4 per evolved strain . . . 22
- Fig. S6 Effect of evolution and assay temperature on phage release rate (conditioning on bacteria growth rate) . . . 23
- Fig. S7 Longevity of waxmoth larvae after injection with *S. marcescens* strains 24
- Fig. S8 Effect of evolution and incubation temperature on bacterial virulence (conditioning on bacteria growth rate) . . . 25
- Fig. S9 Molecular modelling of a *S. marcescens* glycosyltransferase . . . 26
- Fig. S10 Relationship between phage release rates and bacterial yields . . . 27
- Fig. S11 Simulation of qPCR results for different prophage release rates . . . 28
- Fig. S12 Relationship between phage release rates and bacterial growth rates 29

Bibliography **30**

1 Supplementary Methods

1.1 Quantification of phage release rates using qPCR

1.1.1 Culture conditions for the temperature assays

Frozen stocks had been stored at −80 °C in 40 % glycerol, with evolved clones stored in 100-well plates (Bioscreen measurement plates), in randomized order and reference clone stored in microcentrifuge tubes. A preculture step in 400 µl of SPL 1 % at 31 °C was performed by using a cryo-replicator to inoculate evolved clones into a new 100-well plate and by inoculating the reference strain into wells of another plate. After 24 hours, five identical 100-well assay plates containing both the 28 evolved clones of interest and the reference clone were prepared by transferring 40 µl of each preculture into 360 µl of fresh SPL 1 % (1 well per clone, i.e. 29 wells occupied per plate). For the first day of assay, one plate was incubated at 31 °C, two plates at 24 °C and two plates at 38 °C. After 24 hours, clones within a given plate were transferred to 29 previously empty new wells in the same plate (40 µl culture into 360 µl fresh medium). For the second day of assay, the plate from 31 °C was kept at 31 °C, one plate from 24 °C was kept at 24 °C and the other was transferred to 38 °C, and one plate from 38 °C was kept at 38 °C while the other was transferred to 24 °C. After 24 hours, plates were taken for sample processing. Extra wells containing sterile SPL 1 % medium were used on the assay plates to monitor potential contamination during plate handling (which was not observed). The whole experiment was performed twice, starting with the same frozen stocks but with independent precultures.

1.1.2 Sample processing and qPCR runs

At the end of the second day of assay, each of the 29 cultures in each of the 5 assay plates was processed in the following way: 50 µl of native culture sample was transferred to a 96-well PCR plate, while the rest of the culture was placed into a microcentrifuge tube, centrifuged at 13 500 g for 5 min and 50 µl of supernatant was transferred in the 96-well plate, resulting in two paired samples per culture (native and supernatant). Samples from a given assay plate were placed into the same 96-well plate. A DNase treatment was then performed to digest DNA fragments which were not protected inside a bacteria cell or a phage particle. 5 µl of DNase I at 0.1 mg ml^{-1} were added to each sample, followed by an incubation at 37 °C for 30 min. DNA was then released from bacteria cells and potential phage particles by incubating the samples at 95 °C for 30 min after having added 5 µl of EGTA (20 mM, pH 8) in order to hinder DNase I activity. The sample plates were then stored at −20 °C until DNA quantification by qPCR runs.

Quantification of DNA target sequences was performed using prophage-specific primer pairs and one bacterial-gene-specific primer pair (Supplementary Table S2). Preliminary experiments using the reference strain at 31 °C having showed no detectable extra-cellular DNA at least for prophages 2 and 5, six qPCR were runs per 96-well sample plate from this experiment using primers for prophages 1, 3, 4, 6, 7 and for bacterial gene purA2. Runs were performed using CFX Real Time PCR detection system (Bio-Rad laboratories, USA). Amplifications were performed in a final volume

of 10 µl, containing 5 µl of 2 x IQ SYBR Green Supermix (Bio-Rad), 0.5 µl of forward and reverse primers (6 µM each) and 1 µl of undiluted sample. Amplifications for each primer pair were performed on separate qPCR plates, with in-plate calibration samples for each run. Calibration samples were prepared by serial dilution of a stock solution of purified *Serratia marcescens* DNA of known concentration, and ranged in concentration from 10^6 to 1 genome copy per qPCR well, based on the predicted molecular weight of *S. marcescens* chromosome. Experimental and calibration samples were run in triplicates within each qPCR plate. The qPCR reaction used an initial denaturation step lasting 3 min at 95 °C, followed by 41 cycles consisting of denaturation at 95 °C for 10 s, annealing at 61 °C (for all prophage primers) or 56 °C (for bacterial gene primers) for 10 s, and elongation at 72 °C for 30 s. A melt curve analysis was performed at the end of the run to check the quality of the amplified product (from 65 °C to 95 °C, using increments of 0.5 °C and 5 s steps). In-plate calibration samples were used to estimate the efficiency E of the qPCR reaction with the formula $E = -1 + 10^{(-1/\beta)}$ where β is the slope of the linear relationship between Cq values and $\log_{10}$(concentration) for the calibration samples. To test for an effect of potentially undegraded RNA molecules on phage release estimates, some samples were incubated with RNase for 30min just prior to qPCR runs. Estimates of phage release for those samples were similar whether the samples were treated or untreated with RNase prior to qPCR runs, hence data from both RNase-treated and untreated qPCR runs was used for downstream analysis. To check that samples prepared from bacterial cultures did not contain substances which would consistently inhibit the amplification reaction during qPCR runs, we also performed some preliminary tests where we checked that dilution series from a few samples prepared from bacterial cultures could give qPCR amplification efficiency estimates E close to 100%.

1.1.3 Estimation of phage release rates and treatment effects using a Bayesian model

We incorporated into a single Bayesian model the simultaneous estimation of phage release rates and of the effects of assay temperature and evolutionary treatment. To simplify its presentation here, we will first explain the modelling part related to the estimation of release rates for each culture well, based on the Cq values for the native and supernatant samples obtained from qPCR runs with bacterial and prophage primers, before explaining the incorporation of assay and evolutionary treatment effects.

Let $c_{bact,nat}$ be the number of bacterial chromosome copies present in a native sample. The value of $c_{bact,nat}$ is determined from the qPCR run using the bacterial-gene-specific purA2 primers. Let $c_{pro,nat}$ be the number of prophage DNA copies present in the native sample for e.g. prophage KSP20. The value of $c_{pro,nat}$ is determined from the qPCR run using the prophage-specific primers. Let's assume that this prophage is induced and released into phage particles at a release rate a, such that the number of phage particles present in the native sample $c_{phg,nat}$ is related to the number of bacteria cells (i.e. the number of bacteria chromosome copies) by $c_{phg,nat} = a \times c_{bact,nat}$. Since the prophage primers can target the prophage sequence both in the bacterial genome and in phage particles, we have:

$$\begin{aligned} c_{pro,nat} &= c_{bact,nat} + c_{phg,nat} \\ c_{pro,nat} &= c_{bact,nat} + a \times c_{bact,nat} \\ c_{pro,nat} &= (1+a) \times c_{bact,nat} \end{aligned} \tag{1}$$

After centrifugation, we assume most bacteria cells have been pelleted and most phage particles (if any) have remained in suspension. Let k be the concentration factor during centrifugation for this culture, so that $k = (c_{bact,sup}/c_{bact,nat})$ where $c_{bact,sup}$ is the number of bacterial chromosome copies present in the supernatant samples, as determined by qPCR with purA2 primers ($0 \leq k \leq 1$). If $c_{pro,sup}$ is the number of prophage DNA copies in the supernatant sample determined by qPCR with the prophage primers and $c_{phg,sup}$ is the number of phage particles in the supernatant sample, and if we assume $c_{phg,sup} = c_{phg,nat}$ (i.e. we assume that the amount of phage particles pelleted during centrifugation is negligible), we have:

$$\begin{aligned} c_{pro,sup} &= c_{bact,sup} + c_{phg,sup} \\ c_{pro,sup} &= k \times c_{bact,nat} + c_{phg,nat} \\ c_{pro,sup} &= k \times c_{bact,nat} + a \times c_{bact,nat} \\ c_{pro,sup} &= (k+a) \times c_{bact,nat} \end{aligned} \tag{2}$$

Thus, to summarize, the two fundamental equations that relate the four qPCR measurements for a given culture ($c_{bact,nat}$ / $c_{bact,sup}$ / $c_{pro,nat}$ / $c_{pro,sup}$) and the prophage release rate a in this culture are:

$$\begin{aligned} c_{pro,nat} &= (1+a) \times c_{bact,nat} \\ c_{pro,sup} &= \left(\frac{c_{bact,sup}}{c_{bact,nat}} + a \right) \times c_{bact,nat} \end{aligned} \tag{3}$$

We describe below the integrated Bayesian model used to estimate phage release rates based on those two equations and on the Cq data obtained from qPCR runs. Note that in the model description below, all parameters corresponding to DNA concentrations are expressed in number of target copies per qPCR well (copies/well).

The model relates Cq values to DNA concentrations, using plate-specific calibration parameters (calibration samples were present in all qPCR plates). Firstly, the model likelihood component due to the calibration samples is (with n_{cal} being the total number of qPCR wells containing a calibration sample in our dataset):

$$\begin{aligned} \forall i \in \{1 \ldots n_{cal}\}, \qquad c_i^{well} &\sim \text{Poisson}(cal_i) \\ \text{Cq}_i^{pred} &= \alpha_{run_i} + \beta_{run_i} \times \log_{10}\left(c_i^{well}\right) \\ \text{Cq}_i^{cal} &\sim \text{normal}(\mu = \text{Cq}_i^{pred}, \sigma = \sigma_{\text{Cq}}) \end{aligned} \tag{4}$$

where cal_i is the expected number of target copies in the well (between 1 and 10^5

in our experiment), c_i^{well} is the actual number of target copies in the well, $\alpha_{[.]}$ and $\beta[.]$ are calibration parameters describing the relationship between Cq values and DNA concentrations, $run_i \in \{1 \dots n_{runs}\}$ is the index of the qPCR plate corresponding to the calibration sample and Cq_i^{cal} is the observed Cq value for the sample. Note that $\alpha_{[.]}$ and $\beta_{[.]}$ are plate-specific (i.e. they are indexed by run_i) to account for plate variability in the qPCR efficiency, while the parameter σ_{Cq} which accounts for the experimental noise in the observed Cq values is shared across all qPCR runs. Note also that we use a Poisson distribution for c_i^{well} to more accurately describe the sampling process happening when pipetting the template from their preparative tubes into the qPCR wells, especially at low template concentrations.

Secondly, we describe the model likelihood component due to the qPCR wells containing the experimental samples of unknown concentrations prepared from the cultures in the assay plates. For this, we set (with n_{unkn} being the number of qPCR wells with samples of unknown concentration and $cult_i$ the index of the original culture for each unknown sample):

$$\forall i \in \{1 \dots n_{unkn}\}, \qquad unkn_i = \begin{cases} c_{bact,nat,cult_i} & \text{or} \\ c_{bact,sup,cult_i} & \text{or} \\ c_{pro,nat,cult_i} & \text{or} \\ c_{pro,sup,cult_i} & \end{cases} \tag{5}$$

depending on whether the unknown sample is run with purA2 ($c_{bact,.,.}$) or prophage ($c_{pro,.,.}$) primers and whether it is native ($c_{.,nat,.}$) or from supernatant ($c_{.,sup,.}$). The likelihood due to unknown samples is then of the same form as for the calibration samples:

$$\begin{aligned} \forall i \in \{1 \dots n_{unkn}\}, \qquad c_i^{well} &\sim \text{Poisson}(unkn_i) \\ \mathrm{Cq}_i^{pred} &= \alpha_{run_i} + \beta_{run_i} \times \log_{10}\left(c_i^{well}\right) \\ \mathrm{Cq}_i^{cal} &\sim \text{normal}(\mu = \mathrm{Cq}_i^{pred}, \sigma = \sigma_{\mathrm{Cq}}) \end{aligned} \tag{6}$$

The remaining deterministic relationships of the model and the priors used for unknown parameters to estimate are:

$$\begin{aligned} \forall i \in \{1 \dots n_{runs}\}, \qquad \alpha_i &\sim \text{normal}(\mu = 40, \sigma = 10) \\ \beta_i &\sim \text{normal}(\mu = 3.5, \sigma = 2) \\ \sigma_{Cq} &\sim \text{half-Cauchy}(\text{scale} = 2.5) \end{aligned} \tag{7}$$

for the parameters of the qPCR calibration curve for each run (note that σ_{Cq} is shared across all qPCR runs) and:

$$\begin{aligned}
\forall i \in \{1 \dots n_{cultures}\}, \qquad \log_{10}\left(c_{bact,nat,i}\right) &\sim \text{uniform}(0, 20) \\
\log_{10}\left(c_{bact,sup,i}\right) &= \log_{10}\left(k_i\right) + \log_{10}\left(c_{bact,nat,i}\right) \\
\log_{10}\left(c_{pro,nat,i}\right) &= \log_{10}\left(1 + a_i\right) + \log_{10}\left(c_{bact,nat,i}\right) \\
\log_{10}\left(c_{pro,sup,i}\right) &= \log_{10}\left(k_i + a_i\right) + \log_{10}\left(c_{bact,nat,i}\right) \\
\log_{10}\left(k_i\right) &\sim \text{half-Cauchy}(\text{scale} = 2) \\
\log_{10}\left(a_i\right) + 4 &\sim \text{gamma}(\mu = 2, \sigma = 2)
\end{aligned} \tag{8}$$

for the characteristics of a given culture well. Note that here, we assume that the minimum value of release rate a is 10^{-4}, which is approximatively the lower sensitivity threshold predicted for our method when we assume that Cq values are measured with a standard deviation $\sigma_{Cq} \approx 0.48$ (Supplementary Figure S11). We model this as $(\log_{10}(a_i) + 4)$ following a Gamma distribution. In this explanation, we use fixed values for the parameters of the Gamma distribution, but when we will introduce the effect of assay and evolutionary treatment the μ and σ parameters of this Gamma distribution will depend on the treatments.

This model formulation is sufficient to obtain posterior distributions for $\log_{10}(a_i)$ for each culture well i in the assay plates. To model the effect of assay and evolutionary treatment, we extend the model by modifying the parameters of the previous prior for a_i:

$$\log_{10}\left(a_i\right) + 4 \sim \text{gamma}(\mu = 2, \sigma = 2) \tag{9}$$

by:

$$\begin{aligned}
\forall i \in \{1 \dots n_{cultures}\}, \qquad \log_{10}\left(a_i\right) + 4 &\sim \text{gamma}(\mu = \mu_i, \sigma = \sigma_i) \\
\mu_i &\sim \exp\left(\beta_{assay}[assay_i] + \beta_{str}[str_i]\right) \\
\sigma_i &= \sigma_{assay}[assay_i]
\end{aligned} \tag{10}$$

where $assay_i$ is the index of the assay treatment for culture i ($assay_i \in \{1 \dots 5\}$) and str_i is the index of the strain ID for culture i ($str_i \in \{1 \dots 29\}$). The priors for the effect of assay treatments are:

$$\begin{aligned}
\text{Intercept:} \qquad \beta_{assay}[1] &= 1 \\
\forall i \in \{2 \dots 5\}, \qquad \beta_{assay}[i] &\sim \text{normal}\left(\mu = 0, \sigma = 4\right) \\
\forall i \in \{1 \dots 5\}, \qquad \sigma_{assay}[i] &\sim \text{uniform}(0, 10)
\end{aligned} \tag{11}$$

The strain effects include a hierarchical effect of the evolutionary treatment (four levels: three evolution environments plus the reference strain). The priors for the strain and evolutionary treatment effects are:

$$\begin{aligned} \forall i \in \{1 \ldots 29\}, \quad & \beta_{str}[i] \sim \text{normal}\left(\mu = \mu_{evo}[evo_i], \sigma = \sigma_{evo}[evo_i]\right) \\ \forall i \in \{1 \ldots 4\}, \quad & \mu_{evo}[i] \sim \text{normal}\left(\mu = 0, \sigma = 4\right) \\ & \sigma_{evo}[i] \sim \text{uniform}\left(0, 10\right) \end{aligned} \tag{12}$$

where evo_i is the index of the evolutionary treatment for strain i.

1.2 Bayesian implementation of the Cox proportional hazards mixed model

The virulence experiment dataset contained observations for $N = 2182$ individual larvae. For each larvae i, survival time s_i was calculated as the difference between recorded death time and injection time. The survival timeline for all larvae was divided into $T = 20$ intervals, so that the $s_{i,i \in \{1 \ldots N\}}$ values were homogeneously distributed across intervals (i.e. all intervals contained approximatively the same number of death events). Intervals were defined by their boundaries $t_{j,j \in \{1 \ldots T+1\}}$, such that interval j is $[t_j, t_{j+1})$ and is of duration $dt_j = t_{j+1} - t_j$.

The survival data was transformed into a risk variable $Y_i(j)$ and an event count variable $dN_i(j)$ defined for all $i \in \{1 \ldots N\}$ and $j \in \{1 \ldots T\}$ by:

$$Y_i(j) = \begin{cases} 1 & \text{if } s_i > t_j \\ 0 & \text{otherwise} \end{cases} \text{ and } dN_i(j) = \begin{cases} 1 & \text{if } s_i \in [t_j, t_{j+1}) \\ 0 & \text{otherwise} \end{cases} \tag{13}$$

The model assumes:

$$dN_i(j) \sim \text{Poisson}(Y_i(j) \times d\lambda_0(j) \times \exp(\beta z_i) \times dt_j) \tag{14}$$

where $d\lambda_0(j)$ is the increment in the integrated baseline hazard from t_j to t_{j+1} and βz_i is the product of the model parameters and of the covariate values for larva i. The term βz_i corresponds to:

$$\begin{aligned} \beta z_i = \; & \beta_{blk}[blk_i] + \beta_{BM} BM_i + \beta_{OD} OD_i + \beta_{str|incub24}[str_i] \times (1 - incub_i) \\ & + \beta_{str|incub31}[str_i] \times incub_i \end{aligned} \tag{15}$$

where blk_i, BM_i, OD_i, str_i, and $incub_i$ are respectively the replication block, body mass, preculture OD, injected strain ID ($str_i \in \{1 \ldots 29\}$) and incubation temperature (0 for 24 °C, 1 for 31 °C) for larva i. Square brackets indicate indexing of a vector parameter; β_{blk} is a vector containing the replication block effects and $\beta_{str|incub24}$ and $\beta_{str|incub31}$ are vectors containing the strain effects in the 24 °C and 31 °C incubations, respectively. To model the effect of the evolutionary treatment, we set, for $k \in \{1 \ldots 29\}$:

$$\begin{aligned} \beta_{str|incub24}[k] & \sim \text{normal}\left(\mu_{incub24}[evo[k]], \sigma_{incub24}[evo[k]]\right) \\ \beta_{str|incub31}[k] & \sim \text{normal}\left(\mu_{incub31}[evo[k]], \sigma_{incub31}[evo[k]]\right) \end{aligned} \tag{16}$$

where the vector evo allows to map the strain ID and one of the four evolutionary

treatments (three different temperature regimes plus reference strain).

The priors we used were:

$$\begin{aligned}
\beta_{blk}[.] &\sim \text{normal}(\text{mean} = 0, \text{sd} = 10) \\
\beta_{BM} &\sim \text{normal}(0, 10) \\
\beta_{OD} &\sim \text{normal}(0, 10) \\
\mu_{incub24}[.] &\sim \text{normal}(0, 2) \\
\mu_{incub31}[.] &\sim \text{normal}(0, 2) \\
\sigma_{incub24}[.] &\sim \text{uniform}(\text{min} = 0, \text{max} = 10) \\
\sigma_{incub31}[.] &\sim \text{uniform}(0, 10)
\end{aligned} \tag{17}$$

and for all $j \in \{1 \ldots T\}$:

$$\begin{aligned}
d\lambda_0(j) &\sim \text{gamma}(\text{mean} = d\lambda_0^*(j), \text{rate} = c) \\
d\lambda_0^*(j) &= r \times dt_j
\end{aligned} \tag{18}$$

with $c = 0.001$ and $r = 0.1$. We used the first replication block and the effect of the reference strain in the 24 °C incubation as the references:

$$\begin{aligned}
\beta_{blk}[1] &= 1 \\
\mu_{incub24}[anc] &= 1
\end{aligned} \tag{19}$$

We ran four chains in parallel with the JAGS MCMC sampler for 10 000 iterations per chain, of which the first 5000 were discarded as burn-in. Model convergence and chain mixing was assessed by visual examination of trace plots and calculation of $\hat{R}$ values.

1.3 Selection of m6A in non-fully methylated GATC motifs

The method to identify GATC loci which were not fully methylated in our dataset was reported in a companion study (Bruneaux et al., 2021). Briefly, we calculated for each GATC locus the distance between the point defined by its methylated fractions on the plus and minus strand and the point corresponding to full methylation on both strands (1,1). We then defined the set of partially methylated GATC loci of interest as the loci which deviated from the point of full methylation more than four times the average quadratic distance to (1, 1) in at least one sequenced strain.

2 Supplementary Results

2.1 Effect of bacterial growth rate on phage release rate

2.1.1 Statistical model update: adding bacteria growth rate effect

The statistical model described in section 1.1.3 above aims at estimating the effect of evolutionary treatment (via the parameter vector μ_{evo}) and of assay treatment (via the parameter vector β_{assay}) on phage release rates. Conceptually, this model assumes the causal relationships described by the following directed acyclic graph (DAG):

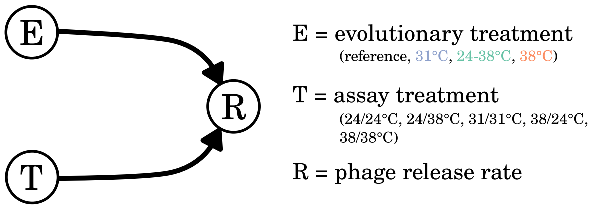

However, both evolutionary treatment (E) and assay temperature (T) have an effect on the bacteria growth rate (Supplementary Figure S1; for the effect of evolutionary treatment on growth rate, see Ketola et al. 2013), which in turn could affect the phage release rates (R) measured in our experiment. The DAG can be updated to reflect these causal assumptions:

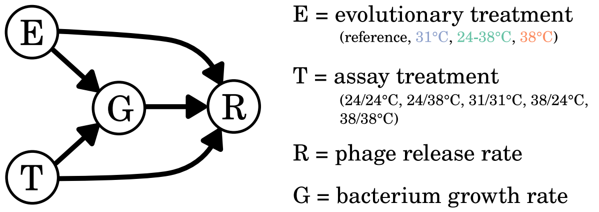

If we assume the causal DAG above, then the statistical model previously described in section 1.1.3 estimates the total effects of evolutionary treatment (E) and assay treatment (T) on phage release (R). It takes into account both their direct effect and their indirect effect mediated via their effect on growth rate (R), without distinguishing between them. We can update the previous statistical model to condition the analysis on bacteria growth rates (G). By doing this, the effect estimates for (E) and (T) in the updated statistical model will only incorporate the direct effect of (E) and (T) on (R), not their indirect effect through (G).

We update the statistical model from section 1.1.3 to condition on bacteria growth rate by modifying the expression for μ_i in Equation (11) as follows:

$$\forall i \in \{1 \ldots n_{cultures}\}, \tag{20}$$

$$\log_{10}(a_i) + 4 \sim \text{gamma}(\mu = \mu_i, \sigma = \sigma_i)$$

$$\mu_i \sim \exp(\beta_{assay}[assay_i] + \beta_{str}[str_i] + \beta_{gr} \times gr[str_i, temp_i])$$

$$\sigma_i = \sigma_{assay}[assay_i]$$

where β_{gr} is the regression coefficient for the effect of bacteria growth rate on phage release, $gr[.,.]$ is a 29×3 array containing the standardized growth rates for each strain (one row per strain) at the temperatures of the second day of the assays (one column per second-day temperature), str_i is the index of the strain ID for culture i ($str_i \in \{1 \ldots 29\}$), and $temp_i$ is the index of the temperature of the final day of the assay for culture i ($temp_i \in \{1, 2, 3\}$, where the indexed temperatures are 24, 31, and 38 °C – we only consider the effect of the temperature during the last day of the two-day assays on bacteria growth rate). We populate the $gr[,.,]$ array with the growth rate values measured in the original dataset of Ketola et al. (2013) (Supplementary Figure S1).

The additional prior for the updated statistical model is:

$$\beta_{gr} \sim \text{normal}\,(\mu = 0, \sigma = 5) \tag{21}$$

Note that the growth rates for the reference strain (coded as $str_i = 29$ in our model implementation) are not measured under the same conditions as the evolved clones in the Ketola et al. (2013) dataset, and we thus consider them as missing values in our model and give them the following priors:

$$\begin{aligned} gr[29,1] &\sim \text{normal}\,(\mu = 0, \sigma = 1) \\ gr[29,2] &\sim \text{normal}\,(\mu = 0, \sigma = 1) \\ gr[29,3] &\sim \text{normal}\,(\mu = 0, \sigma = 1) \end{aligned} \tag{22}$$

The rest of the model is identical to the previously described model where the growth rate variable was not explicitly taken into account.

2.1.2 Model outcome when conditioning on growth rate

Once the updated model conditioning on bacterial growth rate is run, the effects of evolutionary treatment and assay temperature are calculated for the overall mean growth rate (i.e. a $gr[.,.]$ value of 0 since growth rate was standardized). The results are shown in Supplementary Figure S6. Those results can be compared with those shown in Figure 2 in the main text to evaluate if bacteria growth rate is an important mediator of the effect of evolutionary treatment and assay temperature on phage release rate.

The estimates for the effect of evolutionary treatment and assay temperature are almost unchanged when conditioning on growth rate. This suggests that these effects are direct and not mediated via intermediate effects on bacteria growth rate. However, it is important to note that bacteria growth rate as measured in Ketola et al. (2013) is calculated as the maximum growth rate, i.e. the steepest slope of temporal recording of culture optical densities after medium inoculation, and that other characteristics of bacterial growth curves might arguably be influenced by evolutionary treatment and assay temperature and in turn influence phage release rates.

2.2 Effect of bacterial growth rate on bacterial virulence

Similarly to what is presented above for the statistical model describing phage release rates, we can include maximum bacterial growth rate in the model describing bacterial

virulence in waxmoth larvae in order to determine whether the effects of evolutionary treatment and incubation temperature on bacterial virulence are direct or indirect (mediated via their effect on bacterial growth rate).

We update the statistical model from section 1.2 to condition on bacteria growth rate by modifying the expression for βz_i in Equation (15) as follows:

$$\begin{aligned} \beta z_i = \; & \beta_{blk}[blk_i] + \beta_{BM} BM_i + \beta_{OD} OD_i + \beta_{str|incub24}[str_i] \times (1 - incub_i) \\ & + \beta_{str|incub31}[str_i] \times incub_i + \beta_{gr} \times gr[str_i, incub_i + 1] \end{aligned} \tag{23}$$

where β_{gr} is the regression coefficient for the effect of bacteria growth rate on bacterial virulence, $gr[.,.]$ is a 29×2 array containing the standardized growth rates for each strain (one row per strain) at the incubation temperatures of the larvae (one column per temperature), str_i is the index of the strain ID for larva i ($str_i \in \{1 \ldots 29\}$), and $incub_i$ is the incubation temperature (0 for 24 °C, 1 for 31 °C). Again, we populate the $gr[,.,]$ array with the growth rate values measured in the original dataset of Ketola et al. (2013) (Supplementary Figure S1).

The additional priors for the model are:

$$\begin{aligned} \beta_{gr} &\sim \text{normal}\,(\mu = 0, \sigma = 5) \\ gr[29, 1] &\sim \text{normal}\,(\mu = 0, \sigma = 1) \\ gr[29, 2] &\sim \text{normal}\,(\mu = 0, \sigma = 1) \end{aligned} \tag{24}$$

where $gr[29, .]$ are the growth rates of the ancestor, considered as missing values.

The results of this model are shown in Supplementary Figure S8 and can be compared with those from Figure 4 in the main text. The centrality of the posteriors is mostly unchanged while the uncertainty of the estimates becomes larger. All in all, this does not support the hypothesis that the effects of evolutionary treatment and incubation temperature on bacterial virulence would be indirect and mediated via their effect on bacterial growth rate.

2.3 Association between genetic changes and phenotypic traits

The variable loci most clearly associated with both phage release and bacteria virulence in the insect host were the variant *a* and the pooled variants related to galactokinase (pooled variants *b*, *c*, and *h*) and related to glycosyltransferase (pooled variants *e*, *27*, *28*, and *29*) (Figure 7). These genetic variants were located in or close to (≤ 500 bp) genes annotated as transcriptional regulators (molybdenum-dependent transcriptional regulator and transcriptional regulator RcsB involved in motility and capsule and biofilm formation in *E. coli*) and enzymes involved in the cell wall and outer membrane structure and biofilm formation (peptidoglycan synthase, two glycosyltransferases and a cellulose biosynthesis protein BcsG) (Supplementary Table S3). Those genes point towards a potential role for modifications of biofilm structure and of the outer structure of the cellular envelope in modulating phage particle production and virulence in the insect host. In particular, the three independent mutations located in a single glycosyltransferase gene (mutations *28*, *29* and *30*, close to the putative active site of the protein; Supplementary Figure S9; Supplementary Table S3) were observed independently in several strains: three strains evolved at 24-38 °C and one strain evolved at

38 °C. Those independent mutations point to the important role of the outer cellular envelope in the evolution against phage.

Finally, we also noted that haplotype *a*, comprising eleven associated genetic loci, was shared by 5 out of the 8 strains evolved at 38 °C and by the reference strain, but by none of the other sequenced strains. This points to the probable existence of some standing genetic variation at the onset of the experiment, which was then subjected to selection during the experimental evolution (Bruneaux et al., 2021).

2.4 Association between epigenetic changes and phenotypic traits

In addition to nucleotide sequences, the data we obtained from the PacBio SMRT method also provided information about base methylation. In *S. marcescens*, adenosines present in GATC motifs are methylated into m6A by the Dam enzyme at a very high rate (>98% of GATC motifs were methylated on both strands in our dataset; Bruneaux et al. 2021). The remaining GATC motifs can be either hemi-methylated or unmethylated in a cell, and are often variably methylated across cells of a given culture and across strains. Adenosine methylation can influence gene expression by affecting the binding of regulatory proteins to promoter regions of genes (Gomez-Gonzalez et al., 2019) or by affecting transcription speed via increased DNA stability of gene bodies (Riva et al., 2004a,b). Such epigenetic regulation can be maintained across rounds of DNA replication by competitive binding to target DNA between the Dam responsible for methylation and regulatory proteins specific to the same region (Casadesús and Low, 2006, 2013), and can thus be subject to selection.

Among GATC motifs which were not fully methylated in our dataset, no association was found between evolutionary treatments and methylated fractions (Bruneaux et al., 2021). However, we identified adenosines for which changes in methylation level were associated with phenotypic changes in the traits measured here (phage release and virulence in an insect host). For a given phenotypic trait, GATC loci exhibiting both positive and negative correlations between methylated fractions and the trait values could be observed (Figure 8, heatmap panel). Manual curation of the function of the genes associated with GATC motifs related to phenotypic changes showed that many of them were involved in (1) transcription regulation, (2) nutrient capture and transport into the cell, (3) excretion into the outer medium, (4) biofilm formation, adherence or motility, and/or (5) cell envelope structure (including peptidoglycan and lipopolysaccharide biosynthesis) (Figure 8, panel "Probable gene function(s)"). Many of those functional categories have been shown to be critical for pathogen virulence in other bacterial species, in particular for nutrient capture in the challenging host medium (Ren et al., 2018; Liu et al., 2017), for recognition of the host habitat via its nutrient signature (López-Garrido et al., 2015; Krypotou et al., 2019) and for biofilm formation, adherence and motility which have a key role in colonization and successful invasion of the host tissues (Turner et al., 2009; Luo et al., 2017). The numerous candidate genes involved in lipopolysaccharide biosynthesis also suggest that the O antigen, which can classically be involved both in cell recognition by phages and in bacterial virulence in its host (Chart et al., 1989; Li and Wang, 2012), could act as a major player of evolutionary trade-offs between bacterial virulence and resistance to

phage infection.

3 Supplementary tables

Supplementary Table S1: In-silico detection of prophage sequences in *S. marcescens* reference strain genome. Predictions were run on the PHASTER server on 2019-04-21.

Prophage ID	Position in reference strain genome	Size (kb)	Completeness
PP1	521 990-535 146	13.2	incomplete
PP4 (KSP20-like)	1 970 982-2 003 867	32.9	intact
PP5	3 451 823-3 468 581	16.8	intact
PP6	3 914 469-3 946 913	32.4	incomplete
PP7	4 423 686-4 461 768	34.5	intact

Supplementary Table S2: Sequences of the primers used in the qPCR quantification of prophages and chromosomal DNA. The purA2-F/R primers are targeting the chromosomal, non-prophage-related bacterial gene for adenylosuccinate synthetase.

Target	Name	Sequence
Prophage 1	ph1-F	5′-CGGACGTTCTTTCCTCTGCT-3′
	ph1-R	5′-AGCTCTGCAGCGTTATCCAG-3′
Prophage 4 (PP4)	ph4-F	5′-CTTTGGTTCAGGCGTCATGG-3′
	ph4-R	5′-GTAAACCAGTCCCACACGCT-3′
Prophage 5	ph5-F	5′-GCCACATATCCCAGCGTTGA-3′
	ph5-R	5′-ATGGCAAGCCACAGATAGGT-3′
Prophage 6	ph6-F	5′-GTGCCGAAGGAATGGCCTTA-3′
	ph6-R	5′-CTGAAATTGCTTCGCGCCAT-3′
Prophage 7	ph7-F	5′-GTCAAAGGGGTTAAGCTCGC-3′
	ph7-R	5′-GAACAGAACGGCGCACTACA-3′
Bacterial gene	purA2-F	5′-ATGTGGATTACGTGCTGGGC-3′
	purA2-R	5′-CACAGGTATTCGCCGGTTTC-3′

Supplementary Table S3: Genetic variants observed in the 29 sequenced clones (from Bruneaux et al. 2021). Haplotype: letters identify groups of co-occurring mutations. Freq.: minor allele frequency observed among the 28 evolved clones (an asterisk marks loci for which the reference strain carried the minor allele). Distrib.: distribution of minor alleles across the strains evolved in the 31 °C, 24-38 °C and 38 °C treatments.

ID	Haplotype	Freq.	Distrib. (31/fl./38)	Pos. (bp)	Type	Region	Effect	Overlapping or closest (≤500bp) gene: Name	Overlapping or closest (≤500bp) gene: Function
01	f	1/28	0/1/0	31 753	SNP	CDS	non-syn.	DUF3053 domain-containing protein	unknown
02	a	*5/28	0/0/5	40 239	SNP	CDS	non-syn.	HAP resistance protein	Unknown
03	d	1/28	0/1/0	70 546	indel	CDS	frameshift	Icd-like protein	host cell division inhibitor
04		6/28	2/3/1	92 159	indel	non-CDS	-	-	-
05	b	1/28	0/0/1	108 315	indel	non-CDS	-	PTS beta-glucoside transporter	carbohydrate import
06	a	*5/28	0/0/5	131 841	SNP	CDS	non-syn.	cellulose biosynthesis protein BcsG	biofilm?
07		1/28	1/0/0	173 551	indel	non-CDS	-	protoheme IX biogenesis protein HemY	heme metabolism
08	a	*5/28	0/0/5	328 601	SNP	CDS	non-syn.	condensation protein	non-ribosomal peptide synthesis
09		1/28	0/0/1	391 159	indel	CDS	frameshift	RNA chaperone Hfq	regulation of stress transcription factors
10	g	1/28	1/0/0	429 888	indel	non-CDS	-	transcriptional regulator	similar to regulator of E. coli phage Mu
11		4/28	0/4/0	522 878	indel	CDS	frameshift	integrase	prophage DNA Integration/excision
12	b	1/28	0/0/1	751 961	indel	CDS	frameshift	DNA polymerase II	DNA elongation
13	b	1/28	0/0/1	770 532	indel	non-CDS	-	leu operon leader peptide	leucine biosynthesis
14		1/28	1/0/0	914 534	indel	CDS	frameshift	hydroxyacylglutathione hydrolase	lactate metabolism/response to heat
15		1/28	0/1/0	979 119	indel	non-CDS	-	hypothetical protein	unknown
16	f	1/28	0/1/0	1 093 517	indel	CDS	frameshift	competence protein ComEA	cell surface DNA binding
17		1/28	1/0/0	1 185 019	indel	CDS	frameshift	hypothetical protein	unknown
18	c	1/28	0/0/1	1 311 662	indel	CDS	no frameshift	galactokinase	galactose metabolism
19	b	1/28	0/0/1	1 311 735	SNP	CDS	non-syn.	galactokinase	galactose metabolism
20	h	1/28	0/0/1	1 311 996	SNP	CDS	non-syn.	galactokinase	galactose metabolism
21	a	*5/28	0/0/5	1 317 345	SNP	CDS	syn.	Mo-dependent transcriptional regulator	regulation of transcription
22		5/28	1/2/2	1 421 879	indel	non-CDS	-	acyl carrier protein	Fatty acid/polyketide biosynthesis
23	d	1/28	0/1/0	1 609 697	indel	non-CDS	-	transcriptional regulator GalS	regulation of galactose transport/catabolism
24		1/28	0/0/1	1 610 617	indel	CDS	frameshift	galactose/galactoside ABC transporter MglB	galactose import
25		1/28	0/1/0	1 611 529	SNP	CDS	non-syn.	galactose/galactoside ABC transporter MglA	galactose import
26		1/28	1/0/0	1 612 777	indel	CDS	no frameshift	galactose/galactoside ABC transporter MglC	galactose import
27		4/28	0/3/1	1 665 941	indel	CDS	frameshift	glycosyltransferase	LPS biosynthesis
28		1/28	0/1/0	1 670 147	SNP	CDS	non-syn.	glycosyltransferase	LPS biosynthesis
29		2/28	0/1/1	1 670 356	SNP	CDS	non-syn.	glycosyltransferase	LPS biosynthesis
30	e	1/28	0/1/0	1 670 370	SNP	CDS	non-syn.	glycosyltransferase	LPS biosynthesis
31	a	*5/28	0/0/5	1 861 227	indel	non-CDS	-	putative transcriptional regulator	regulation of transcription
32		1/28	0/0/1	2 144 682	indel	non-CDS	-	hypothetical protein	unknown

(Continued on next page.)

ID	Haplotype	Freq.	Distrib. (31/fl./38)	Pos. (bp)	Type	Region	Effect	Overlapping or closest (≤500bp) gene	
								Name	Function
33		1/28	0/1/0	2 282 483	indel	non-CDS	-	MATE family efflux transporter	Na+/H+ driven multidrug efflux pump
34	h	1/28	0/0/1	2 353 326	indel	CDS	frameshift	fumarase C (iron independent)	TCA cycle
35	b	1/28	0/0/1	2 384 093	indel	CDS	frameshift	HlyD (haemolysin secretion system)	haemolysin/cutinase excretion
36	a	*5/28	0/0/5	2 456 338	SNP	CDS	non-syn.	peptidoglycan synthase	peptidoglycan biosynthesis
37		2/28	2/0/0	2 466 586	SNP	CDS	non-syn.	MmgE/PrpD family protein	propionate metabolism/TCA cycle?
38	e	1/28	0/1/0	2 941 884	indel	non-CDS	-	VOC family protein	unknown
39		2/28	2/0/0	3 161 361	SNP	CDS	syn.	serine/threonine protein kinase	regulation of cell processes
40	e	1/28	0/1/0	3 408 594	indel	non-CDS	-	nucleoside diphosphate hydrolase	regulation of cell processes
41	a	*5/28	0/0/5	3 477 366	SNP	CDS	non-syn.	transcriptional regulator RcsB	capsule synthesis/cell division/biofilm/motility
42		*0/28	-	3 600 509	indel	non-CDS	-	phospholipid-binding lipoprotein MlaA	Outer membrane maintenance
43	a	*5/28	0/0/5	3 607 617	SNP	CDS	non-syn.	heme exporter protein CcmB	cytochrome c biogenesis
44	a	*5/28	0/0/5	3 869 219	SNP	CDS	syn.	cytochrome c	energy metabolism
45		1/28	1/0/0	4 025 724	indel	non-CDS	-	acetyl-CoA carboxylase alpha subunit	lipid metabolism
46		1/28	1/0/0	4 337 062	indel	non-CDS	-	tRNA-Phe	translation
47		1/28	0/0/1	4 362 753	indel	non-CDS	-	glycoporin	carbohydrate import
48	c	1/28	0/0/1	4 845 837	indel	CDS	frameshift	peptidylprolyl isomerase	protein folding chaperone
49	g	1/28	1/0/0	4 872 989	indel	CDS	frameshift	short chain dehydrogenase	oxidoreductase
50	d	1/28	0/1/0	4 924 755	SNP	CDS	non-syn.	threonine dehydratase	amino acid metabolism
51	a	*5/28	0/0/5	5 010 850	indel	CDS	frameshift	deacetylase	LPS biosynthesis
52	a	*5/28	0/0/5	5 010 868	SNP	CDS	non-syn.	deacetylase	LPS biosynthesis

4 Supplementary figures

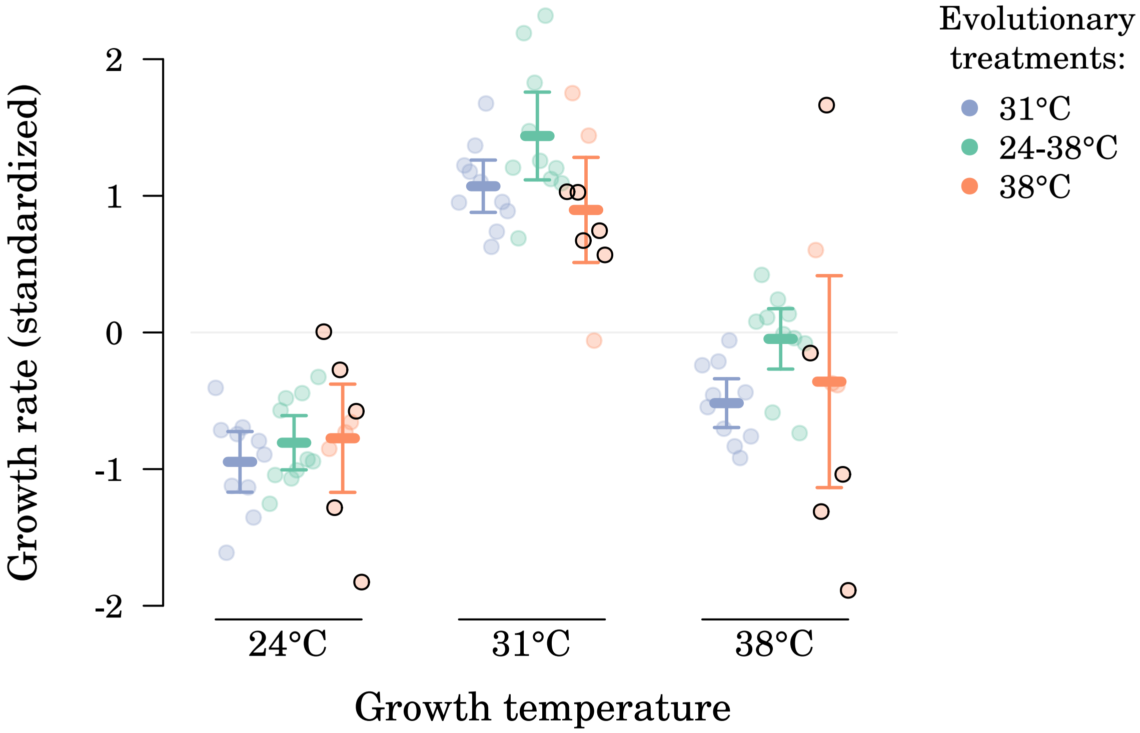

Supplementary Figure S1: Growth rates for the sequenced evolved clones (n = 28) measured at three different temperatures. Each evolved strain is represented by one measurement at a given temperature. Data points circled in black correspond to the strains with the highest phage release rates at 21/21 °C and 31/31 °C in Supplementary Figure S5. Mean value $\pm 1.96 \times$ s.e. is shown for each group of strains from the same evolutionary treatment within a growth temperature. Growth rate values were taken from the original dataset from Ketola et al. (2013) and standardized to a mean of 0 and standard deviation of 1.

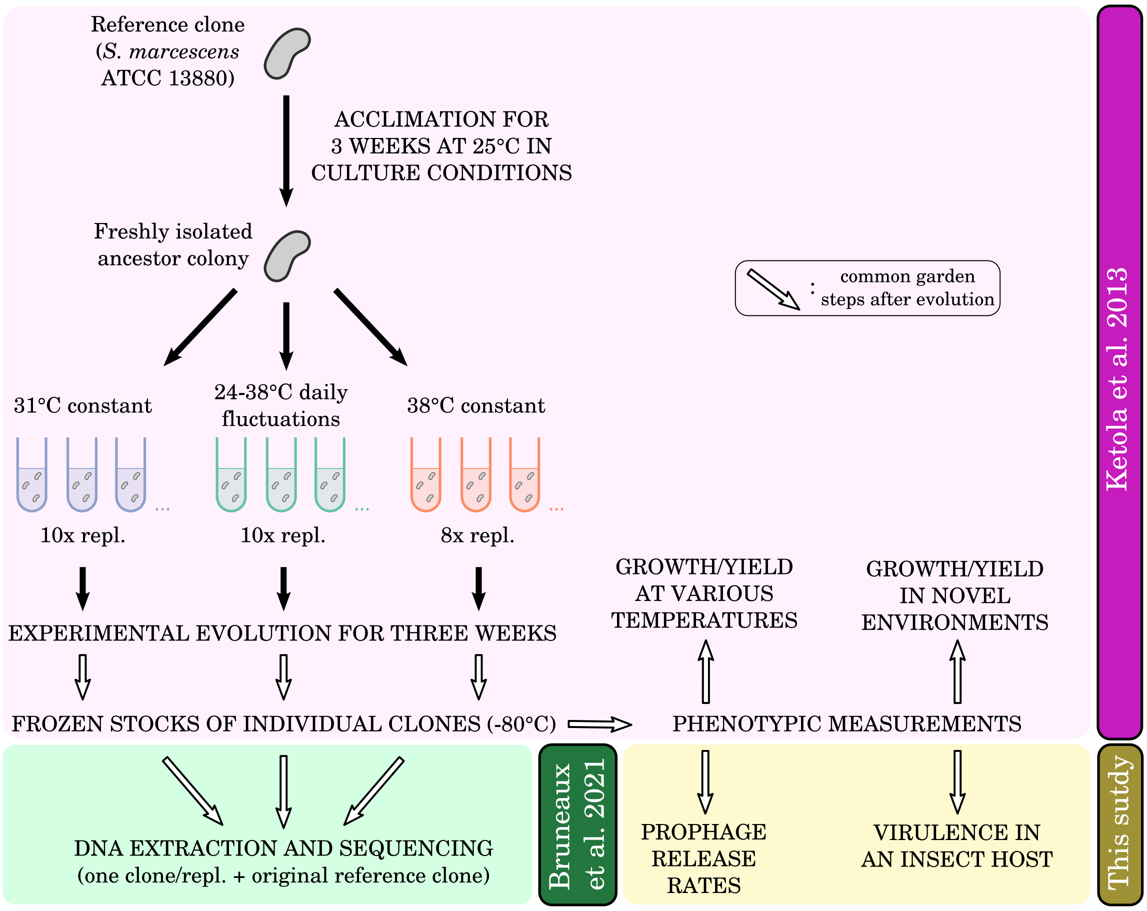

Supplementary Figure S2: Setup of the evolution experiment from which clones were isolated and of downstream measurements. One randomly selected clone per evolved population was used for sequencing. Open arrows after experimental evolution indicates steps where evolved clones were grown under common garden conditions. Details of the evolution experiment are available in Ketola et al. (2013).

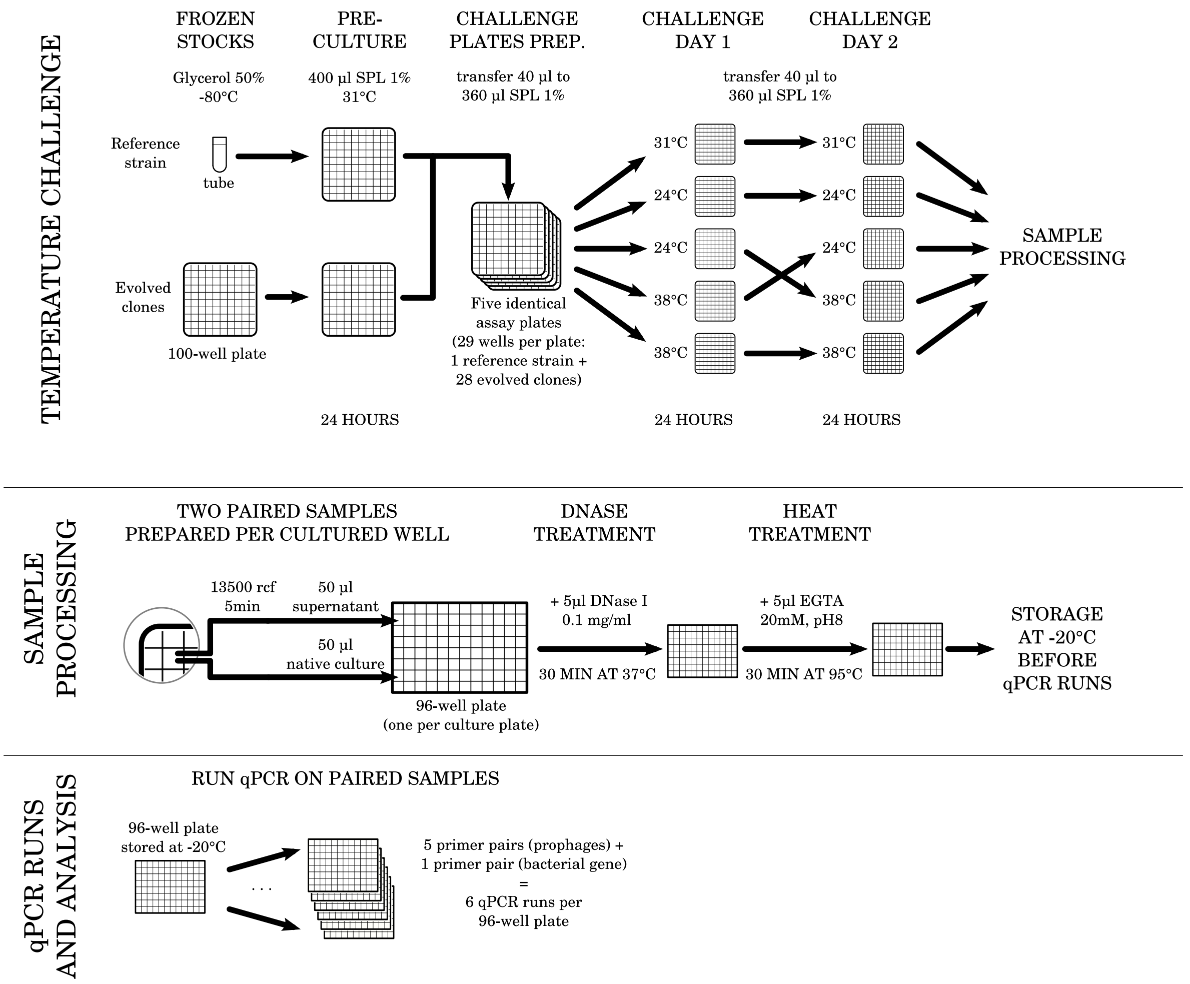

Supplementary Figure S3: Overview of the experimental protocol used for the prophage induction assays. The prophage primers used in the qPCR runs were for prophages 1, 3, 4, 6 and 7, after preliminary experiments with the reference strain showed no detectable amount of extra-cellular DNA for prophages 2 and 5.

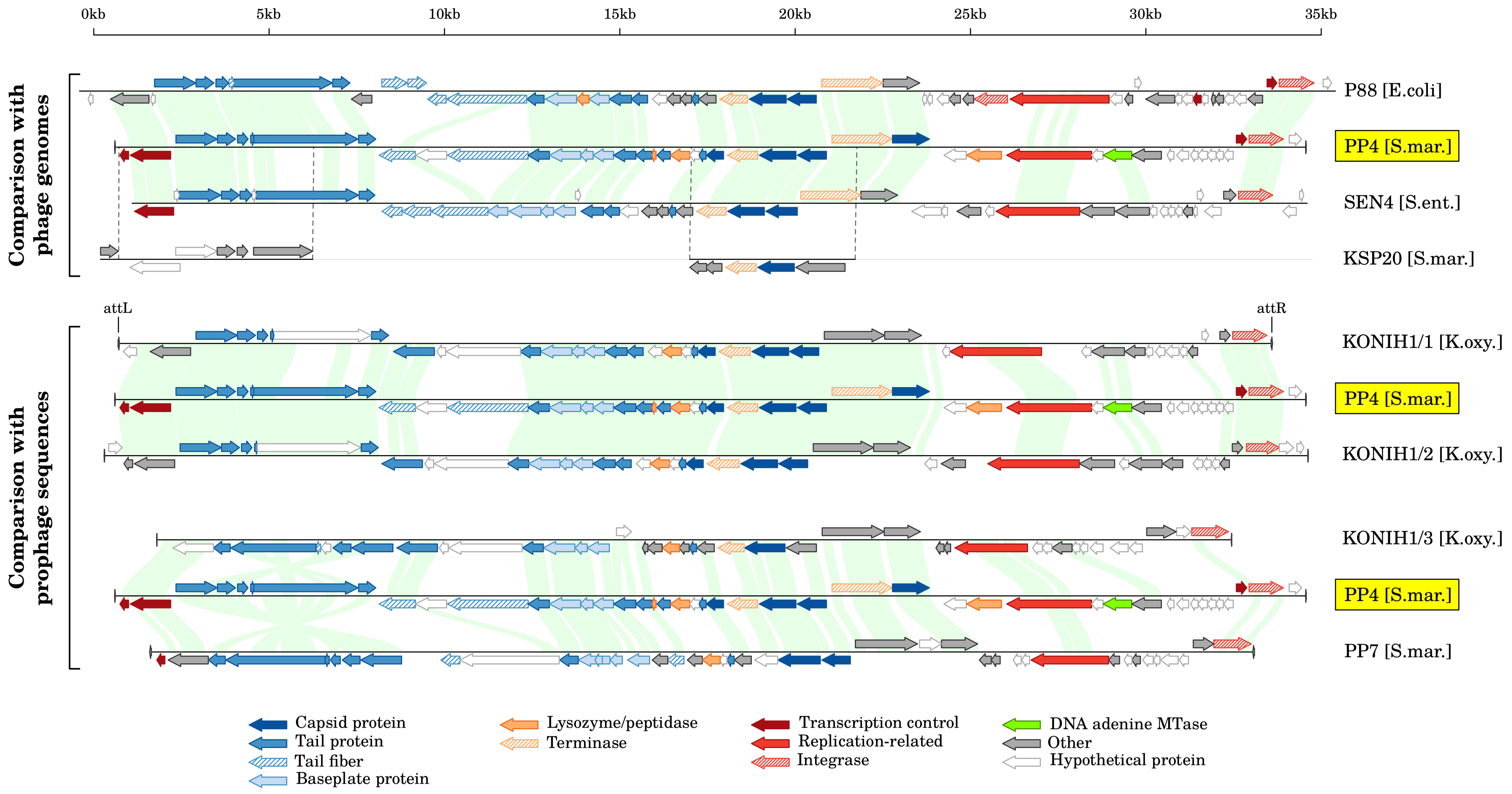

Supplementary Figure S4: Comparison of prophage PP4 with related (pro)phages. P88 and SEN4 infect *Escherichia coli* and *Salmonella enterica*, respectively. KSP20 infects *S. marcescens* (only two sequence fragments available). KONIH1/1-3 are three prophages identified in the genome of a *Klebsiella oxytoca* strain. PP7 is another prophage candidate identified in the genome of the *S. marcescens* strain used in our study but for which no phage release was detected. Matches found with `tblastx` with an e-value $\leq 10^{-80}$ are highlighted in green. For KSP20, those matches cover the entire areas delimited by vertical dashed lines. See details about the search for sequences related to PP4 in Methods.

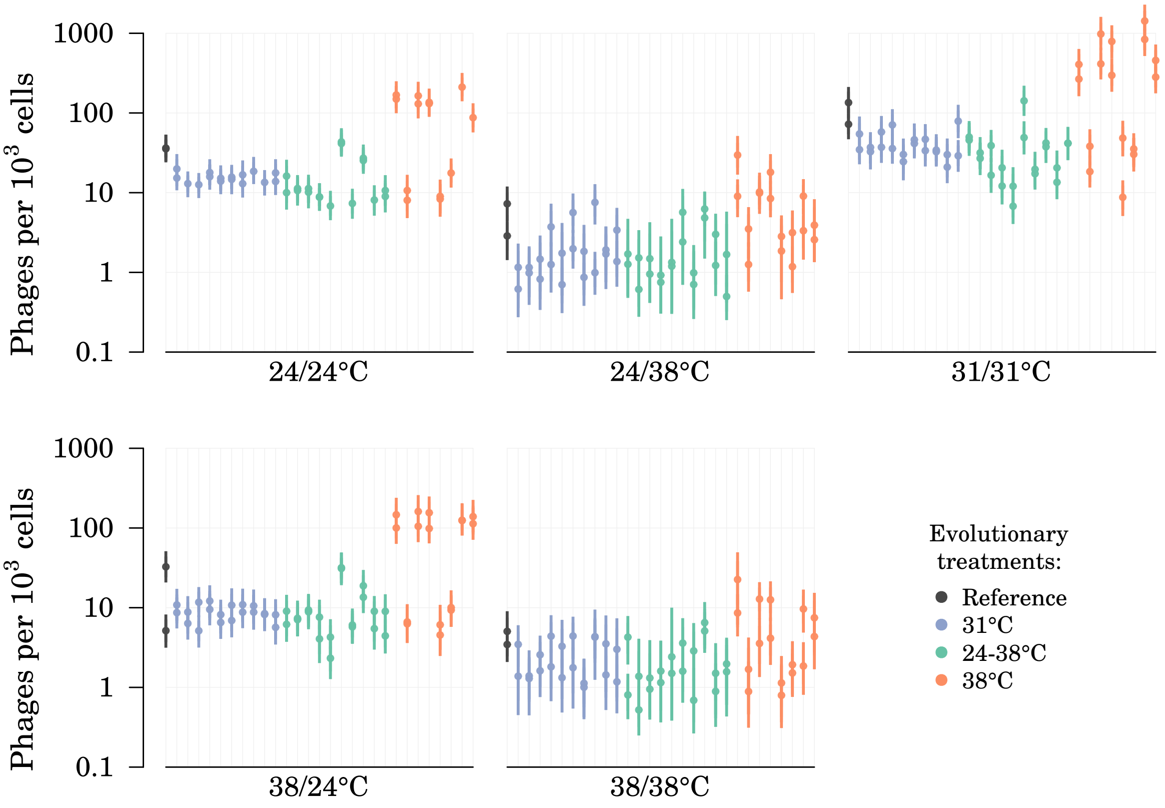

Supplementary Figure S5: Estimated release rates of phage PP4 per evolved strain per assay. For each strain in each assay, two replicate measurements are available in most cases. Estimated prophage release rates are shown as posterior mean and 95% credible interval for each measurement.

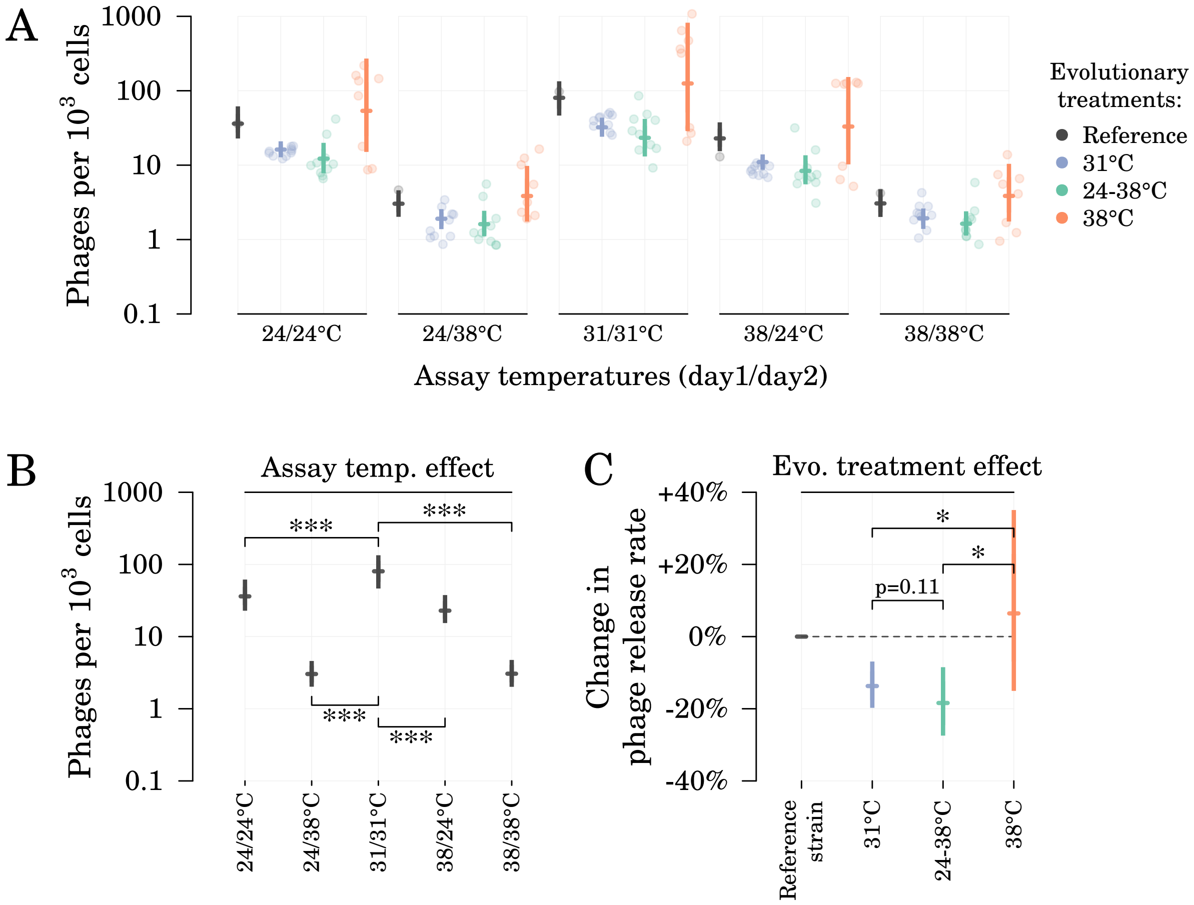

Supplementary Figure S6: Effect of evolutionary treatment and assay temperatures on the release rates of prophage PP4 when conditioning on growth rate. Assays lasted two days and assay temperatures are given as day1/day2. This figure can be compared with Figure 2 in the main text to evaluate the importance of bacteria maximum growth rate in mediating the effect of evolutionary treatment and of assay temperature on phage release rates, which turns out to be negligible (i.e. almost none of the effect of evolutionary treatment and of assay temperature on phage release rates is mediated via their effect on bacteria maximum growth rate). (A) Posteriors of the model-estimated mean for each treatment/assay combination. Points are estimated phage release rates for each of the 29 sequenced clones. (B) Estimates of the assay temperature effects and (C) estimates of the evolutionary treatment effects, with the reference strain used as a reference point. Posteriors are shown as median and 95% credible interval. One-sided Bayesian p-values for pairwise comparisons denoted by * ($p < 0.05$) and *** ($p < 0.001$).

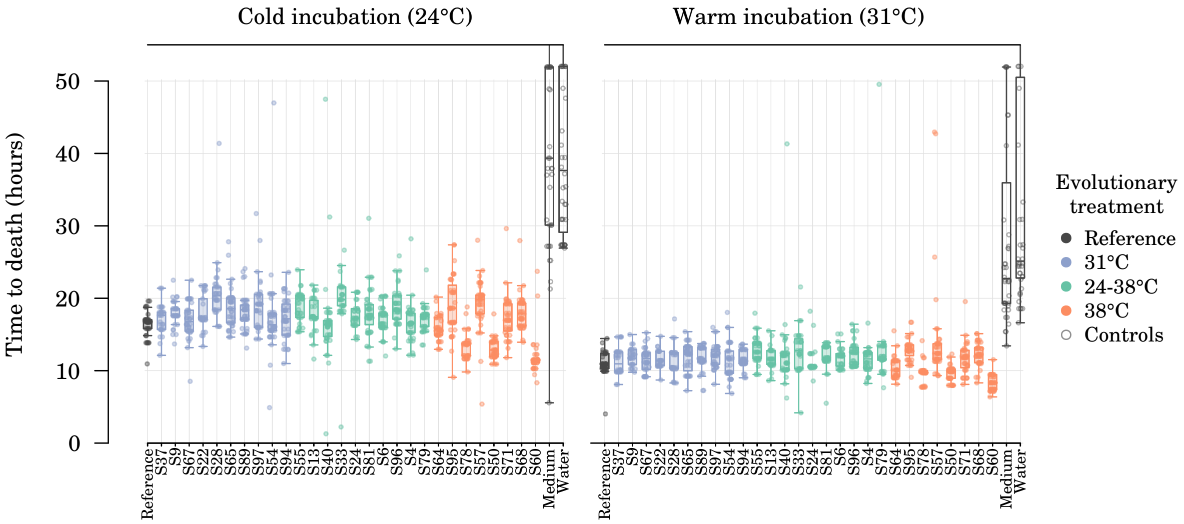

Supplementary Figure S7: Longevity of waxmoth larvae at two incubation temperatures after injection with experimental *Serratia marcescens* strains. Each vertical lane shows larvae injected with a given strain. The Medium and Water lanes show control larvae injected with sterile medium and sterile water, respectively. Longevity is corrected for the effect of replication blocks. Dots are individual larvae. Boxplots center line, median; box limits, upper and lower quartiles; whiskers extend to the most extreme data points which are no more than 1.5 times the interquartile range away from the box.

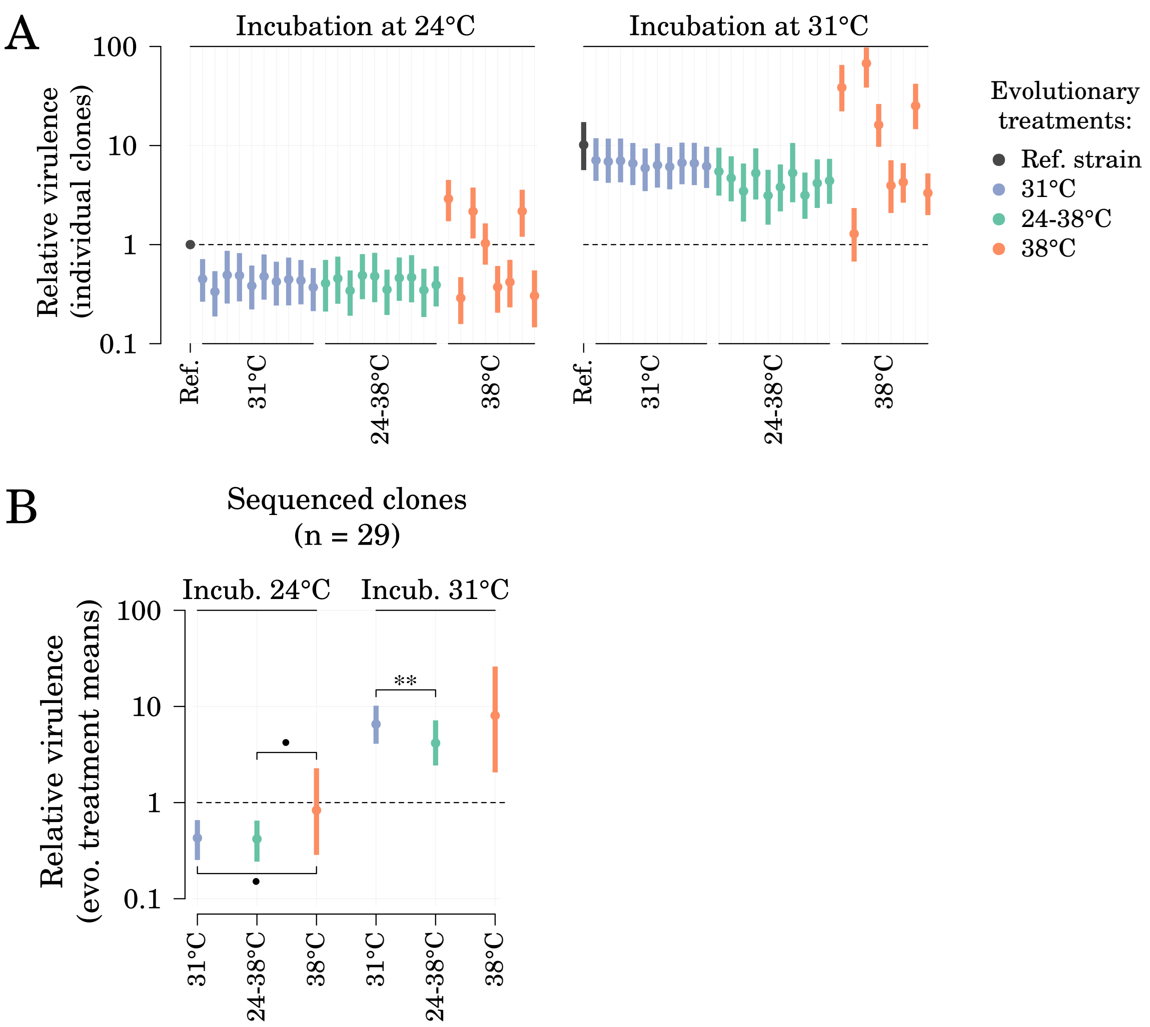

Supplementary Figure S8: Effect of evolutionary treatment and incubation temperatures on bacterial virulence when conditioning on growth rate. This figure can be compared with Figure 4 in the main text to evaluate the importance of bacteria maximum growth rate in mediating the effect of evolutionary treatment and of assay temperature on bacterial virulence. The centrality of the posteriors is mostly unchanged, but estimtates uncertainty increases slightly. (A) Relative virulence of individual sequenced clones, measured as relative hazards estimated from a Bayesian implementation of a Cox proportional-hazards model. All virulence estimates are relative to the virulence of the reference strain in incubation at 24 °C (denoted by a broken horizontal line) and are corrected for the effects of injection batch, larval body mass and optical density of injected cultures. (B) Mean relative virulence per evolutionary treatment and per incubation temperature as estimated by the model ($\exp(\mu_{evo})$) ($n = 29$ sequenced clones).

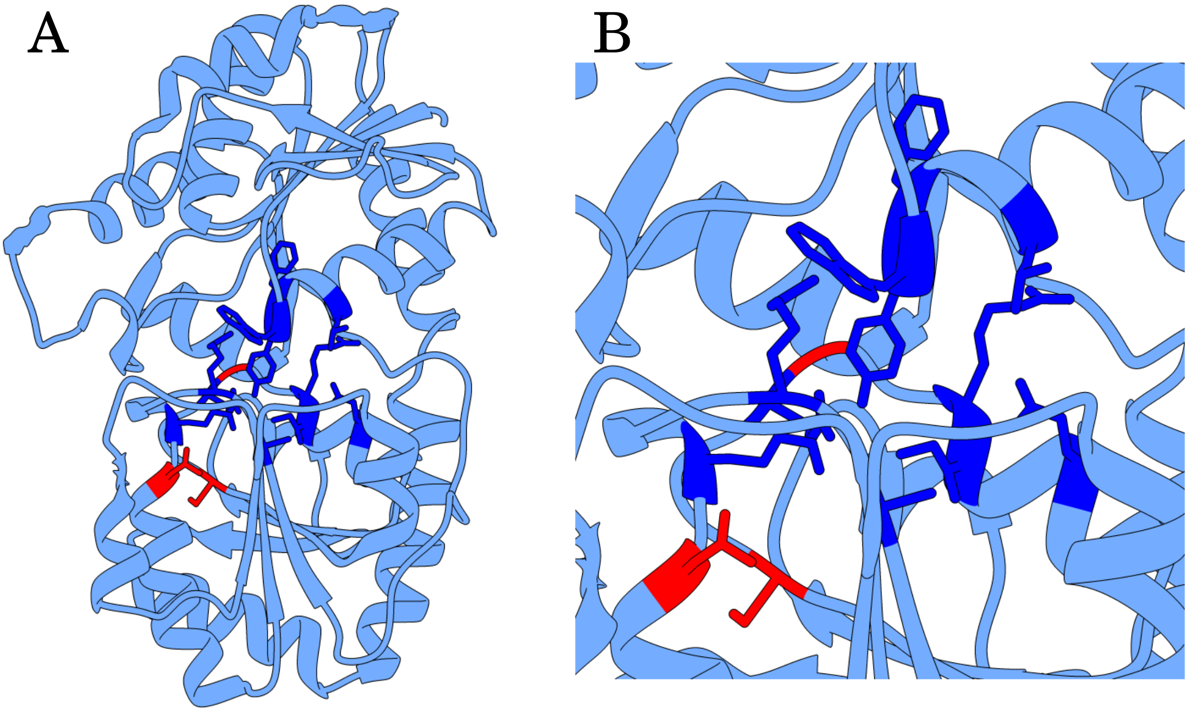

Supplementary Figure S9: Location of observed amino acid substitutions in the predicted structure of a glycosyltransferase of *Serratia marcescens* (GenBank record QSP20947.1). The predicted protein is 380 amino-acid long and its structure was modelled using the Phyre2 online server (Kelley et al. 2015; "intense mode" run submitted on 2015-10-26). A) Overview of the tertiary structure of the complete monomer; B) close-up of the putative active site. Blue, amino-acids which have a side chain close to the ligand site, based on a protein of related structure (PDB 3mbo, Parsonage et al. 2010); red, amino-acids which exhibited three independent mutations in the evolved strains in our experiment (mutations *28*, *29* and *30* in Supplementary Table S3).

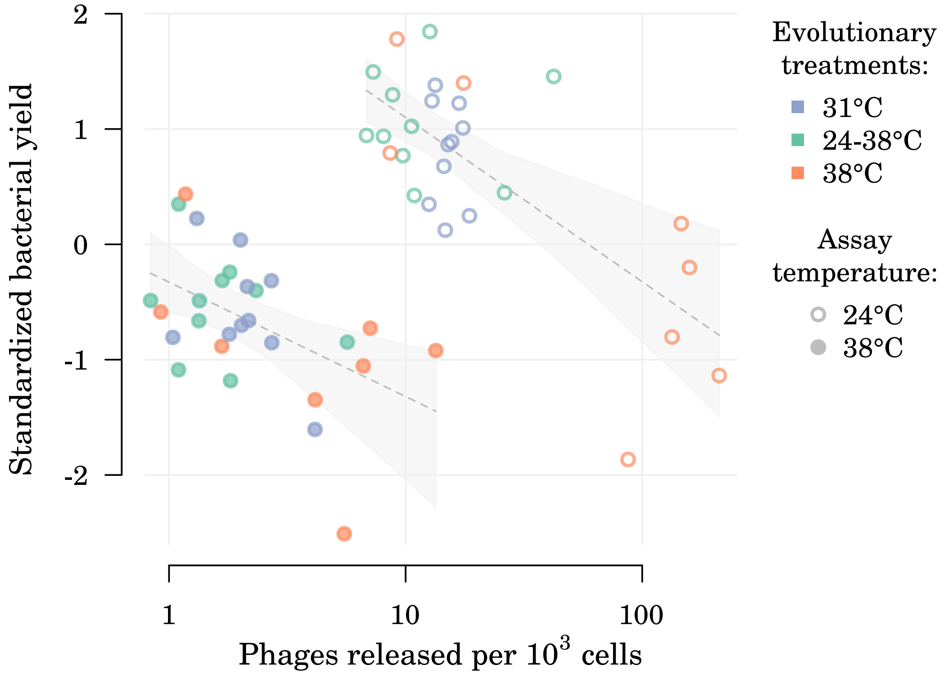

Supplementary Figure S10: Relationship between bacterial yield and phage release rate for the sequenced evolved strains used in this study ($n = 28$). Yield values at 24 °C and 38 °C were taken from the original dataset from Ketola et al. (2013) and standardized to a mean of 0 and standard deviation of 1. Correlations between phage release rates and bacterial yields are: Spearman's $\rho = -0.51$, $p = 0.006$ at 24 °C and $\rho = -0.46$, $p = 0.015$ at 38 °C. A similar figure showing the relationship between bacterial growth rate and phage release rate is available as Supplementary Figure S12. Trend lines within each assay temperature are added for visual support only and are built using ordinary least squares regressions (95%-enveloppes built using 500 bootstraps).

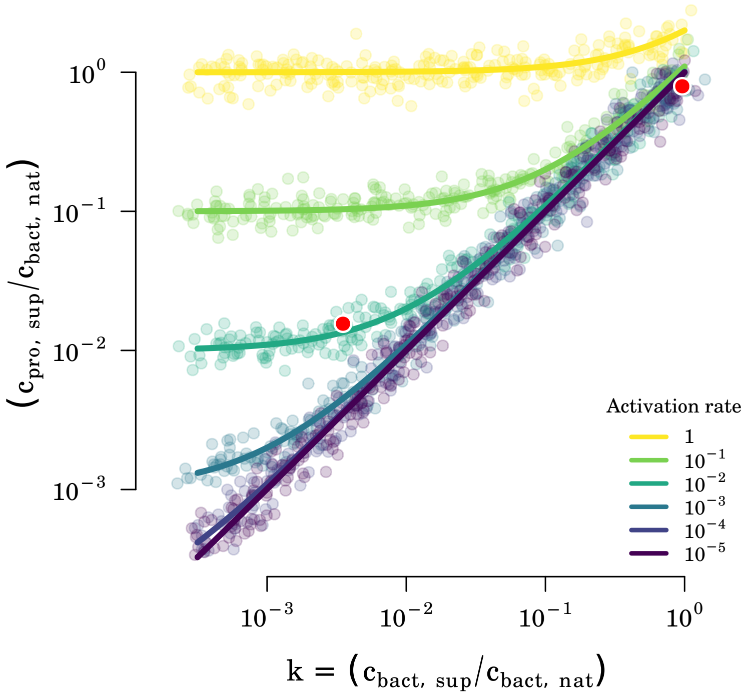

Supplementary Figure S11: Simulation of qPCR results for different prophage release rates a and different centrifugation concentration factors k. $c_{bact,nat}$, $c_{bact,sup}$ and $c_{pro,sup}$ are the qPCR quantifications of the number of DNA copies for bacterial gene in native and supernatant samples and for prophage gene in supernatant samples, respectively. The colored lines show the predicted trajectories of $c_{pro,sup}/c_{bact,nat}$ versus $c_{bact,sup}/c_{bact,nat}$ from native samples (top-right corner) towards supernatant samples (to the left) as the centrifugation concentration factor k decreases (i.e. as supernatant samples are more and more impoverished in bacteria cells). The shape of the trajectories depends on the release rate of the prophage, i.e. on how many phage particles are present per bacteria cells in the native sample. The colored dots matching the colored predicted trajectories represent simulations of qPCR estimations which would be obtained as the centrifugation removes more and more bacteria cells from the supernatant, assuming a precision of the Cq values $\sigma_{cq} = 0.48$ and triplicates qPCR measurements for each culture well, as was done in our experiment. As can be seen on the figure, the sensitivity threshold to detect phage particles decreases as the depletion of bacteria cells becomes more complete. However, even at k values of 10^{-3}, release rates of 10^{-4} and lower are not distinguishable from the absence of release. The red dots represent the results for a hypothetical culture, with the top-right dot representing the native sample and the bottom left dot representing the supernatant sample.

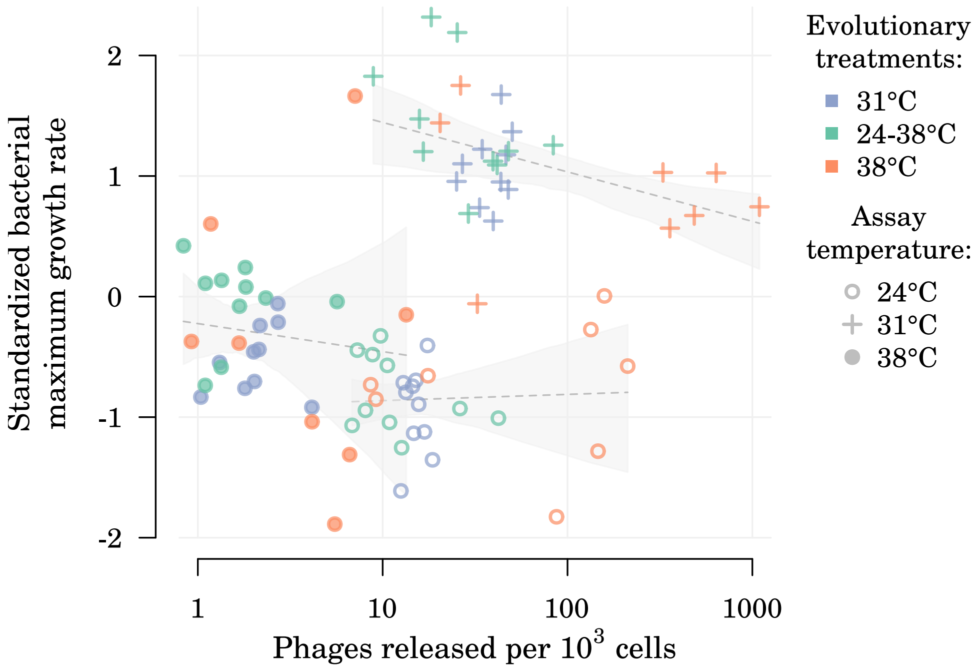

Supplementary Figure S12: Relationship between bacterial maximum growth rate and phage release rate for the sequenced evolved strains used in this study ($n = 28$). Maximum growth rate values at 24 °C, 31 °C, and 38 °C were taken from the original dataset from Ketola et al. (2013) and standardized to a mean of 0 and standard deviation of 1. Correlations between phage release rates and bacterial maximum growth rates are: Spearman's $\rho = -0.03$, $p = 0.86$ (at 24 °C), $\rho = -0.46$, $p = 0.015$ (at 31 °C), and $\rho = -0.15$, $p = 0.44$ at 38 °C.

Bibliography (for Supplementary Materials)

Bruneaux M, Kronholm I, Ashrafi R, Ketola T. 2021. Roles of adenine methylation and genetic mutations in adaptation to different temperatures in *Serratia marcescens*. *Epigenetics*. 0(0):1–21.

Casadesús J Low DA. 2006. Epigenetic gene regulation in the bacterial world. *Microbiology and Molecular Biology Reviews*. 70(3):830–856.

Casadesús J Low DA. 2013. Programmed heterogeneity: epigenetic mechanisms in bacteria. *Journal of Biological Chemistry*. 288(20):13929–13935.

Chart H, Row B, Threlfall EJ, Ward LR. 1989. Conversion of *Salmonella enteritidis* phage type 4 to phage type 7 involves loss of lipopolysaccharide with concomitant loss of virulence. *FEMS Microbiology Letters*. 60(1):37–40.

Gomez-Gonzalez PJ, Andreu N, Phelan JE, de Sessions PF, Glynn JR, Crampin AC, Campino S, Butcher PD, Hibberd ML, Clark TG. 2019. An integrated whole genome analysis of *Mycobacterium tuberculosis* reveals insights into relationship between its genome, transcriptome and methylome. *Scientific Reports*. 9(1):5204.

Kelley LA, Mezulis S, Yates CM, Wass MN, Sternberg MJE. 2015. The Phyre2 web portal for protein modeling, prediction and analysis. *Nature Protocols*. 10(6):845–858.

Ketola T, Mikonranta L, Zhang J, Saarinen K, Örmälä AM, Friman VP, Mappes J, Laakso J. 2013. Fluctuating temperature leads to evolution of thermal generalism and preadaptation to novel environments. *Evolution*. 67:2936–2944.

Krypotou E, Scortti M, Grundström C, Oelker M, Luisi BF, Sauer-Eriksson AE, Vázquez-Boland J. 2019. Control of bacterial virulence through the peptide signature of the habitat. *Cell Reports*. 26(7):1815–1827.e5.

Li J Wang N. 2012. The gpsX gene encoding a glycosyltransferase is important for polysaccharide production and required for full virulence in *Xanthomonas citri* subsp. *citri*. *BMC Microbiology*. 12(1):31.

Liu W, Huang L, Su Y, Qin Y, Zhao L, Yan Q. 2017. Contributions of the oligopeptide permeases in multistep of *Vibrio alginolyticus* pathogenesis. *MicrobiologyOpen*. 6 (5).

Luo M, Yang S, Li X, Liu P, Xue J, Zhou X, Su K, Xu X, Qing Y, Qiu J, Li Y. 2017. The KP1_4563 gene is regulated by the cAMP receptor protein and controls type 3 fimbrial function in *Klebsiella pneumoniae* NTUH-K2044. *PLoS ONE*. 12(7).

López-Garrido J, Puerta-Fernández E, Cota I, Casadesús J. 2015. Virulence gene regulation by L-arabinose in *Salmonella enterica*. *Genetics*. 200(3):807–819.

Parsonage D, Newton GL, Holder RC, Wallace BD, Paige C, Hamilton CJ, Dos Santos PC, Redinbo MR, Reid SD, Claiborne A. 2010. Characterization of the N-acetyl-α-D-glucosaminyl L-malate synthase and deacetylase functions for bacillithiol biosynthesis in *Bacillus anthracis*. *Biochemistry.* 49(38):8398–8414.

Ren W, Rajendran R, Zhao Y, Tan B, Wu G, Bazer FW, Zhu G, Peng Y, Huang X, Deng J, Yin Y. 2018. Amino acids as mediators of metabolic cross talk between host and pathogen. *Frontiers in Immunology.* 9.

Riva A, Delorme MO, Chevalier T, Guilhot N, Hénaut C, Hénaut A. 2004a. Characterization of the GATC regulatory network in *E. coli*. *BMC genomics.* 5(1):48.

Riva A, Delorme MO, Chevalier T, Guilhot N, Hénaut C, Hénaut A. 2004b. The difficult interpretation of transcriptome data: the case of the GATC regulatory network. *Computational Biology and Chemistry.* 28(2):109–118.

Turner KH, Vallet-Gely I, Dove SL. 2009. Epigenetic control of virulence gene expression in *Pseudomonas aeruginosa* by a LysR-type transcription regulator. *PLoS Genetics.* 5(12):e1000779.
